# The archaeal KEOPS complex possesses a functional Gon7 homolog and has an essential function independent of the cellular t^6^A modification level

**DOI:** 10.1002/mlf2.12051

**Published:** 2023-01-08

**Authors:** Pengju Wu, Qi Gan, Xuemei Zhang, Yunfeng Yang, Yuanxi Xiao, Qunxin She, Jinfeng Ni, Qihong Huang, Yulong Shen

**Affiliations:** ^1^ State Key Laboratory of Microbial Technology, CRISPR and Archaea Biology Research Center, Microbial Technology Institute Shandong University Qingdao China

**Keywords:** archaea, DNA homologous recombination repair, Gon7, KEOPS, t^6^A modification

## Abstract

Kinase, putative Endopeptidase, and Other Proteins of Small size (KEOPS) is a multisubunit protein complex conserved in eukaryotes and archaea. It is composed of Pcc1, Kae1, Bud32, Cgi121, and Gon7 in eukaryotes and is primarily involved in N^6^‐threonylcarbamoyl adenosine (t^6^A) modification of transfer RNAs (tRNAs). Recently, it was reported that KEOPS participates in homologous recombination (HR) repair in yeast. To characterize the KEOPS in archaea (aKEOPS), we conducted genetic and biochemical analyses of its encoding genes in the hyperthermophilic archaeon *Saccharolobus islandicus*. We show that aKEOPS also possesses five subunits, Pcc1, Kae1, Bud32, Cgi121, and Pcc1‐like (or Gon7‐like), just like eukaryotic KEOPS. Pcc1‐like has physical interactions with Kae1 and Pcc1 and can mediate the monomerization of the dimeric subcomplex (Kae1‐Pcc1‐Pcc1‐Kae1), suggesting that Pcc1‐like is a functional homolog of the eukaryotic Gon7 subunit. Strikingly, none of the genes encoding aKEOPS subunits, including Pcc1 and Pcc1‐like, can be deleted in the wild type and in a t^6^A modification complementary strain named TsaKI, implying that the aKEOPS complex is essential for an additional cellular process in this archaeon. Knock‐down of the Cgi121 subunit leads to severe growth retardance in the wild type that is partially rescued in TsaKI. These results suggest that aKEOPS plays an essential role independent of the cellular t^6^A modification level. In addition, archaeal Cgi121 possesses dsDNA‐binding activity that relies on its tRNA 3ʹ CCA tail binding module. Our study clarifies the subunit organization of archaeal KEOPS and suggests an origin of eukaryotic Gon7. The study also reveals a possible link between the function in t^6^A modification and the additional function, presumably HR.

## Impact statement

We show that the Kinase, putative Endopeptidase, and Other Proteins of Small size (KEOPS) complex in archaea contains five subunits just as that in eukaryotes. We identified that one of the two Pcc1 paralogs in archaea is a functional homolog of the eukaryotic Gon7. Strong evidence supports the fact that KEOPS in thermophilic archaea plays an essential role in a fundamental cellular process, presumably homologous recombination (HR), independent of the t^6^A modification level. We propose a link between t^6^A modification and HR as well as telomere maintenance of KEOPS. The study provides insights into the structure and evolution of the KEOPS and will be helpful for further understanding the functional mechanism of the evolutionarily conserved complex.

## INTRODUCTION

Kinase, putative Endopeptidase, and Other Proteins of Small size (KEOPS) is a multisubunit protein complex conserved in eukaryotes and archaea^1^, ^2^. Its subunits Pcc1, Kae1, Bud32, Cgi121, and eukaryotic fifth subunit Gon7 form a linear architecture as Gon7‐Pcc1‐Kae1‐Bud32‐Cgi121 or Cgi121‐Bud32‐Kae1‐Pcc1‐Pcc1‐Kae1‐Bud32‐Cgi121, in which Pcc1 serves as a dimerization module, while Gon7 interacts with Pcc1 to eliminate the Pcc1 dimer^3^, ^4^, ^5^. The primary role of KEOPS is threonylcarbamoyl (TC) modification at A37 of NNU anticodon transfer RNAs (tRNAs), yielding N^6^‐threonylcarbamoyl adenosine (t^6^A)^6^. t^6^A modification is critical for stabilizing the tRNA anticodon loop structure and base pairing of the anticodon with the corresponding codon for the enhancement of translation fidelity in all three domains of life^6^, ^7^. In humans, defects in t^6^A modification due to Gon7 mutants lead to the Galloway–Mowat syndrome, which is characterized by infantile onset of microcephaly and central nervous system abnormalities^8^, ^9^.

In eukaryotic cytoplasm and archaea, the KEOPS complex catalyzes t^6^A modification in association with Sua5 in two steps. First, Sua5 synthesizes the intermediate TC‐AMP and then KEOPS transfers TC to tRNA^10^. In the second step, the 3ʹ CCA ssRNA binding subunit Cgi121 recruits tRNAs to the KEOPS complex, while Pcc1, Kae1, and Bud32 recognize the NNU tRNA together^11^. During TC transfer by KEOPS, the TC‐transferase Kae1 functions as the active center and the kinase/ATPase subunit Bud32 stimulates the activity of Kae1^12^. However, the precise role of Gon7 in tRNA modification is still unclear.

Besides t^6^A modification, KEOPS also plays a role in homologous recombination (HR) repair and telomere maintenance in budding yeast, both of which are critical for genome integrity and cell survival^1^, ^13^, ^14^. End resection of DNA double‐strand breaks (DSBs) is an essential step in HR repair^15^, ^16^. The Mre11‐Rad50‐Xrs2/Mre11‐Rad50‐NBS1(MRX/N) complex, in conjunction with Sae2/CtIP, initiates the resection, which is followed by two distinct pathways mediated by Exo1/EXO1 and Dna2/DNA2‐Sgs1/BLM, respectively, generating a long 3ʹ‐tail of single‐stranded DNA (ssDNA)^17^, ^18^, ^19^, ^20^. Recent studies reported that in knockout yeast of the KEOPS subunits, the production of ssDNA near DNA break ends and HR efficiency were reduced, indicating that the KEOPS complex is a novel important player in DSB end resection or its regulation^13^. Additionally, the deletion of the KEOPS genes in yeast led to telomere shortening, and depletion of KEOPS subunits in the *cdc13‐1* mutant strain reduced the amount of telomeric ssDNA, indicating that the complex is also involved in the maintenance of telomeres by regulating telomere replication and recombination^1^. Although the mechanism of KEOPS complex in t^6^A modification is relatively clearly known, the precise role of the KEOPS complex in DSB repair and telomere maintenance is largely unexplored. Interestingly, the functions of KEOPS in HR and telomere maintenance are independent of the t^6^A modification^13^, ^21^.

Archaea harbor a eukaryotic‐like DNA transaction system and serve as good models for studying the biology of archaea and the evolution of life. In fact, many eukaryotic‐like HR proteins of archaea, especially Mre11 and Rad50 and their complexes, have served as excellent models for elucidating the structural and functional mechanisms for the eukaryotic counterparts^22^, ^23^, ^24^. In thermophilic archaea, all key proteins involved in HR are essential^25^, ^26^, indicating that HR plays a fundamental role in DNA repair for the survival of these archaea. Similarly, archaeal KEOPS (aKEOPS) has been used as a model for the structural and biochemical studies of eukaryotic KEOPS^10^, ^11^, ^12^, ^27^. Nevertheless, so far, genetic analysis has been carried out only in a halophilic euryarchaeon *Haloferax volcanii* for some of the KEOPS subunits^28^. Interestingly, in this euryarchaeon, the fused *kae1‐bud32* gene is essential, and so was the *cgi121*. In contrast, *pcc1* (encoding the putative dimerizing unit of KEOPS) was not essential. Deletion of *pcc1* led to pleiotropic phenotypes, including decreased growth rate and increased cellular DNA content^28^, implying a failure in chromosome segregation and/or DNA repair. Whether the DSB repair function of KEOPS is conserved or not in archaea and eukaryotes and its relationship with other functions, tRNA modification and telomere maintenance, remain unclear to date.

In this work, we carried out in vivo and biochemical analyses of the KEOPS subunit in *Saccharolobus islandicus* (formally *Sulfolobus islandicus*) REY15A, a thermophilic model crenarchaeon close to the ancestral eukaryotes. We show that one Pcc1 homolog, Pcc1‐like, is the fifth subunit and functional Gon7 homolog of aKEOPS. We further show that each of the five genes encoding aKEOPS subunits (including the gene annotated as *pcc1‐like*) is essential in the wild type and a strain complemented with a bacterial t^6^A modification system, suggesting that KEOPS may have additional and essential functions in *S. islandicus*. Further biochemical analysis suggests that the Pcc1‐Kae1 subcomplex and Cgi121 are the main dsDNA binding players, and three key conserved residues of Cgi121 involved in tRNA 3ʹ CCA tail binding also contribute to dsDNA binding, revealing a possible link between two functions of the complex: t^6^A modification and HR. Finally, a model for the evolutional scenario of t^6^A modification systems is proposed.

## RESULTS

### Pcc1‐like is the fifth subunit of the KEOPS complex in archaea

**Figure 1:**
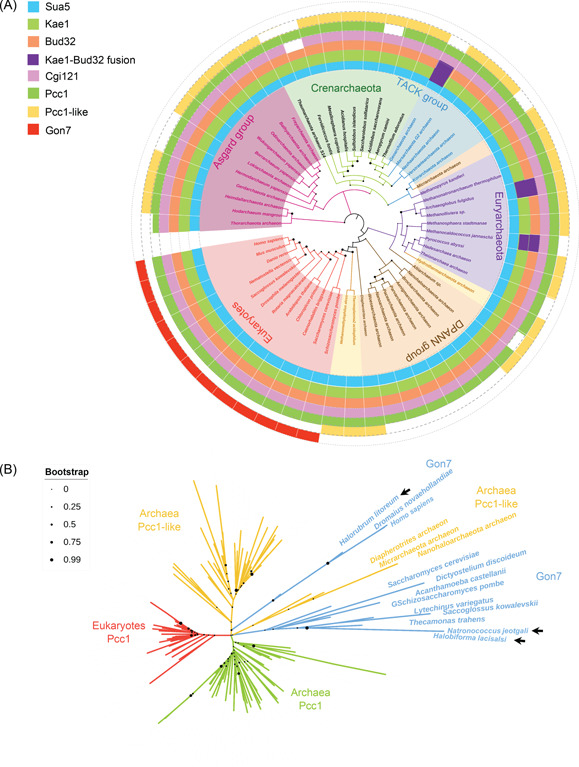
Distribution and phylogenetic analysis of Sua5 and KEOPS subunits in archaea and eukaryotes. (A) Distribution of Sua5 and KEOPS subunits. The phylogenetic tree was constructed based on the amino acid sequences of 59 Kae1 proteins from representative species of each archaea phylum and eukaryotes using the Neighbor‐Joining method. The homologs were identified by protein–protein BLAST and protein–nucleotide BLAST in NCBI (https://blast.ncbi.nlm.nih.gov). If no homolog of KEOPS subunits was found by BLAST, genome context analysis was conducted using ribosome genes, which co‐occurred with most KEOPS subunits, or XTP dephosphorylase, which co‐occurred with the Asgard cgi121, as the references. (B) Phylogenetic analysis of Pcc1 and Gon7 homologs. The unrooted maximum likelihood tree of Pcc1, Pcc1‐like, and Gon7 homologs was constructed based on 126 sequences from 82 species of archaea and eukaryotes. Filled circles at the nodes indicate bootstrap values of 1000 replicates in different sizes. The arrows indicate Gon7 sequences as a domain of the proteasome‐activating nucleotidases (WP_007139789.1 from *Halobiforma lacisalsi* AJ5, and ELY65446.1 from *Natronococcus jeotgali* DSM 18795), or MutS (WP_008364526.1 from *Halorubrum litoreum*) of halobacteria of euryarchaea. The sequences are available upon request. KEOPS, Kinase, putative Endopeptidase, and Other Proteins of Small size.

Four subunits of KEOPS, Pcc1, Kae1, Bud32, and Cgi121, are highly conserved in archaea and eukaryotes^3^. Although the fifth subunit Gon7 was identified in yeast and humans^1^, ^2^, ^5^, it is still unclear whether aKEOPS contains a counterpart of Gon7. To find a putative Gon7 ortholog in archaea, we searched for the distribution of the homologs of Sua5 and KEOPS subunits in all archaeal and representative eukaryotic phyla. It is confirmed that only Sua5, Pcc1, Kae1, and Bud32, the minimal components for t^6^A modification, and Cgi121 are conserved in all archaea and eukaryotes at the amino acid sequence level (Figure 1A). Consistent with the previous report, no sequence homolog of Gon7 is found in archaea as a single domain protein^5^. However, in rare cases, in some halophilic archaea, Gon7 is present as a domain of a proteasome‐activating nucleotidase, such as WP_007139789.1 from *Halobiforma lacisalsi* AJ5, and ELY65446.1 from *Natronococcus jeotgali* DSM 18795, or MutS (e.g., WP_008364526.1 from *Halorubrum litoreum*) (Figure 1B). Interestingly, many archaea harbor two Pcc1 paralogs denoted Pcc1 and Pcc1‐like, which are encoded by SiRe_1278 and SiRe_1701, respectively, in *S. islandicus* REY15A^29^. Pcc1 is more widely distributed than Pcc1‐like. The distribution of Pcc1‐like is also found in most superphyla, with the least appearance in the DPANN superphylum (Figure 1A). Sequence alignment and phylogenetic analysis of Pcc1, Pcc1‐like, and Gon7 reveal that Pcc1 has a higher evolution rate than the other subunits (not shown) and archaeal Pcc1‐like and eukaryotic Gon7 homologs form a separate lineage that is distal to both archaeal Pcc1 and eukaryotic Pcc1 (Figure 1B). These results confirm that one of the two Pcc1 paralogs in archaea, Pcc1‐like, is a possible Gon7 homolog of eukaryotes.

To further confirm whether Pcc1‐like is involved in the formation of aKEOPS in vivo, we conducted affinity purification and mass spectrometry analysis by overexpression of the tagged subunits in *S. islandicus* REY15A. As shown in Table 1, both Pcc1 and Pcc1‐like were co‐purified with Kae1 and Kae1‐Bud32 from the Kae1 and Kae1‐Bud32 overexpression cells, respectively, while Kae1 and Pcc1‐like were co‐purified with Pcc1 from the Pcc1 overexpression cells. Intriguingly, Sua5, which catalyzes the synthesis of t^6^A intermediate TC‐AMP, was also co‐purified with Kae1 and Kae1‐Bud32 from their overexpression cells, respectively, indicating that Sua5 might interact with the KEOPS complex in vivo. Next, in vitro pull‐down analysis showed that Pcc1‐like was able to pull down Pcc1 and Kae1 (Figure S1A). Therefore, we provide strong evidence suggesting that Pcc1‐like is the fifth subunit of KEOPS in *Sulfolobales*.

**Table 1:** Sua5 and KEOPS subunits identified from co‐purified proteins of various KEOPS subunits expressed in *Saccharolobus islandicus* by mass spectrometry.

Bait protein	Component	Mass (Da)	Score	Sequence^a^	Coverage (%)
His‐Kae1					
	Sua5	39,611	236	7 (7)	86
	Kae1	36,323	26,851	35 (31)	33
	Pcc1	10,523	103	3 (3)	51
	Pcc1‐like	9138	124	2 (1)	28
His‐Pcc1					
	Kae1	36,323	205	3 (3)	11
	Pcc1	10,523	198	3 (2)	35
	Pcc1‐like	9138	34	1 (1)	13
Kae1/His‐Bud32					
	Sua5	39,611	7543	25 (24)	72
	Kae1	36,323	1126	17 (15)	45
	Bud32	26,899	5229	13 (12)	46
	Pcc1	10,523	42	2 (1)	34
	Pcc1‐like	9138	15	2 (1)	22

^a^ Total number of sequences matched. The number of matches above the significance threshold is shown in parentheses.

### Pcc1‐like eliminates Pcc1‐mediated dimerization of the Pcc1‐Kae1 subcomplex in vitro

A linear four‐subunit organization of Pcc1:Kae1:Bud32:Cgi121 was proposed for the KEOPS complex of a euryarchaeon *Methanococcus jannaschii* and budding yeast *Saccharomyces cerevisiae* ^3^, ^4^. To date, there has been no study on the subunit interaction of KEOPS from *Crenarchaea*. To determine the inter‐subunit relationship of the crenarchaeal KEOPS, each subunit (except for Pcc1) of the KEOPS from *S. islandicus* was expressed in and purified from *Escherichia coli* and a pull‐down assay was performed. As shown in Figure S1B,C, Bud32 was pulled down by His‐tagged Kae1 and MBP‐tagged Cgi121, respectively, confirming that there are interactions between Bud32 and Kae1 as well as between Bud32 and Cgi121. Since Pcc1 is insoluble when it is expressed in *E. coli* alone, we co‐expressed Pcc1 and Kae1 in *E. coli*. His‐Pcc1 was able to pull down Kae1, confirming that Pcc1 interacts with Kae1 (Figure S1A, middle). Therefore, linear subunit organization of Pcc1:Kae1:Bud32:Cgi121 should also be the case for KEOPS from *Crenarchaea*.

**Figure 2:**
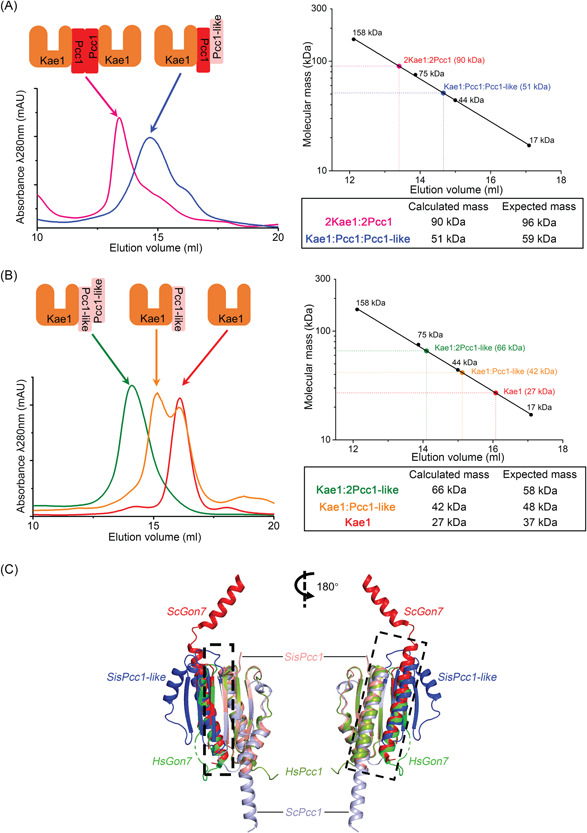
Subunit interaction and complex formation of Pcc1, Pcc1‐like, and Kae1 revealed by pull‐down and size exclusion chromatography (SEC). (A) SEC profiles of Kae1/Pcc1 (pink) and Kae1/Pcc1 + Pcc1‐like (blue). (B) SEC profiles of Kae1/Pcc1‐like (Pcc1‐like 10‐fold excess, green), Kae1/Pcc1‐like (Kae1 five‐fold excess, orange), and Kae1 (red). The molecular masses of subcomplexes were calculated based on the protein standards (right). (C) Structural modeling of *Saccharolobus islandicus* (*Sis*) Pcc1:Pcc1‐like and its superposition with *Saccharomyces cerevisiae* (*Sc*) Pcc1:Gon7 and *Homo sapiens* (*Hs*) Pcc1:Gon7^30^. The modeling was performed using the online tool AlphaFold2_advanced (https://colab.research.google.com/github/sokrypton/ColabFold/blob/main/beta/AlphaFold2_advanced.ipynb) based on the structures of the *Pyrococcus furiosus* homologs. Structure alignment was conducted using the cealign (CE algorithm) in PyMOL.

However, our analysis of the interaction between Kae1 and Pcc1‐like indicates that the organization of Pcc1, Pcc1‐like, and Kae1 from *S. islandicus* was not in a linear mode. It was reported that the KEOPS complex of *M. jannaschii* and *S. cerevisiae* exists as a dimeric structure mediated by a Pcc1 dimer, each monomer of which interacts with a separate Kae1 molecule^27^. The fifth subunit of eukaryotic KEOPS Gon7 interacts with Pcc1 and abolishes this dimerization^4^. To understand whether Pcc1‐like functions as Pcc1 or an evolutionary intermediate corresponding to Gon7, we examined subcomplex formation among Pcc1, Pcc1‐like, and Kae1 by size exclusion chromatography (SEC). As shown in Figure 2A, the peak of the coexpressed Kae1‐Pcc1 subcomplex is corresponding to a 90 kDa complex, which was close to a 2:2 subcomplex with the expected molecular mass of 96 kDa. Coexpressed Kae1‐Pcc1‐like subcomplex was eluted at a peak of 66 kDa, corresponding to a Kae1:2 Pcc1‐like subcomplex with a predicted molecular mass of 58 kDa when Pcc1‐like was about 10‐fold higher than Kae1 (Figure 2B), indicating that Pcc1‐like could not mediate the dimerization of the Kae1‐Pcc1‐like subcomplex (ca. 96 kDa). When Kae1 was in excess (five folds of Pcc1‐like), the stoichiometry of the Kae1‐Pcc1‐like subcomplex became 1:1, with an observed mass of 42 kDa, close to the expected mass of 48 kDa (Figure 2B). Notably, when 1.5‐fold Pcc1‐like was added to the mixture of the 2Kae1:2Pcc1 subcomplex, the 2:2 peak disappeared and a 51 kDa peak was formed (Figure 2A), suggesting a 1:1:1 stoichiometry of Kae1:Pcc1:Pcc1‐like. It should be noted that the elution volume of the Kae1:Pcc1:Pcc1‐like subcomplex was between those of Kae1:2 Pcc1‐like and Kae1:Pcc1‐like, but not close to that of Kae1:2 Pcc1‐like, suggesting that there was a putative conformational change after the ternary subcomplex was formed. These results suggest that Pcc1 mediates the dimerization of Kae1‐Pcc1, while Pcc1‐like disrupts the 2Kae1:2Pcc1 subcomplex and stabilizes the Kae1–Pcc1 subcomplex, same as that in eukaryotic Pcc1 and Gon7 in eukaryotes^4^, ^11^. In *M. jannaschii*, there was a small conformational change when the 2:2 Kae1–Pcc1 subcomplex changed to a 1:2 one, implying a transition state during Pcc1‐like evolution^27^. Our structural superposition shows that Pcc1‐like matches with yeast and human Gon7 proteins with two β sheets and one α helix at the interaction surface of Gon7‐Pcc1 (Figure 2C)^30^, reinforcing that Pcc1‐like is a potential Gon7 homolog. Hence, we propose that archaea Pcc1‐like is the putative functional and structural homolog of Gon7.

### All the genes encoding aKEOPS subunits are essential for cell viability in *S. islandicus*

In eukaryotes, KEOPS plays a role in t^6^A modification, HR repair, and telomere maintenance, while only the t^6^A modification function of KEOPS was found in archaea^10^, ^13^, ^21^. Telomeres were absent in archaea probably due to their circular chromosomes^31^. Therefore, to determine whether aKEOPS also functions in HR repair, it is important to understand the original functions and mechanisms of KEOPS. To address the role of aKEOPS, we attempted to knock out each of its encoding genes in the crenarchaeon using the genome‐editing method based on the endogenous CRISPR‐Cas systems^32^. These included the genes coding for Pcc1‐like, Pcc1, Kae1, Bud32, and Cgi121. We found that none of these genes could be deleted after more than three attempts (Figure S2), consistent with a previous report that they are essential in the *S. islandicus* M16.4 strain^33^. In bacteria, the genes involved in t^6^A synthesis could not be knocked out in nearly all species, possibly due to their essential role in the t^6^A modification^34^. In budding yeast, deletion of the KEOPS subunit genes resulted in a t^6^A modification defect and very slow growth^35^. Therefore, this raised a question of whether the observed essentiality of KEOPS genes in *S. islandicus* could be attributed to the t^6^A modification function or other fundamental ones that are essential for cell viability such as HR repair in *Sulfolobales* ^25^, ^26^.

### A bacterial t^6^A biosynthesis system can be introduced into *S. islandicus*

**Figure 3:**
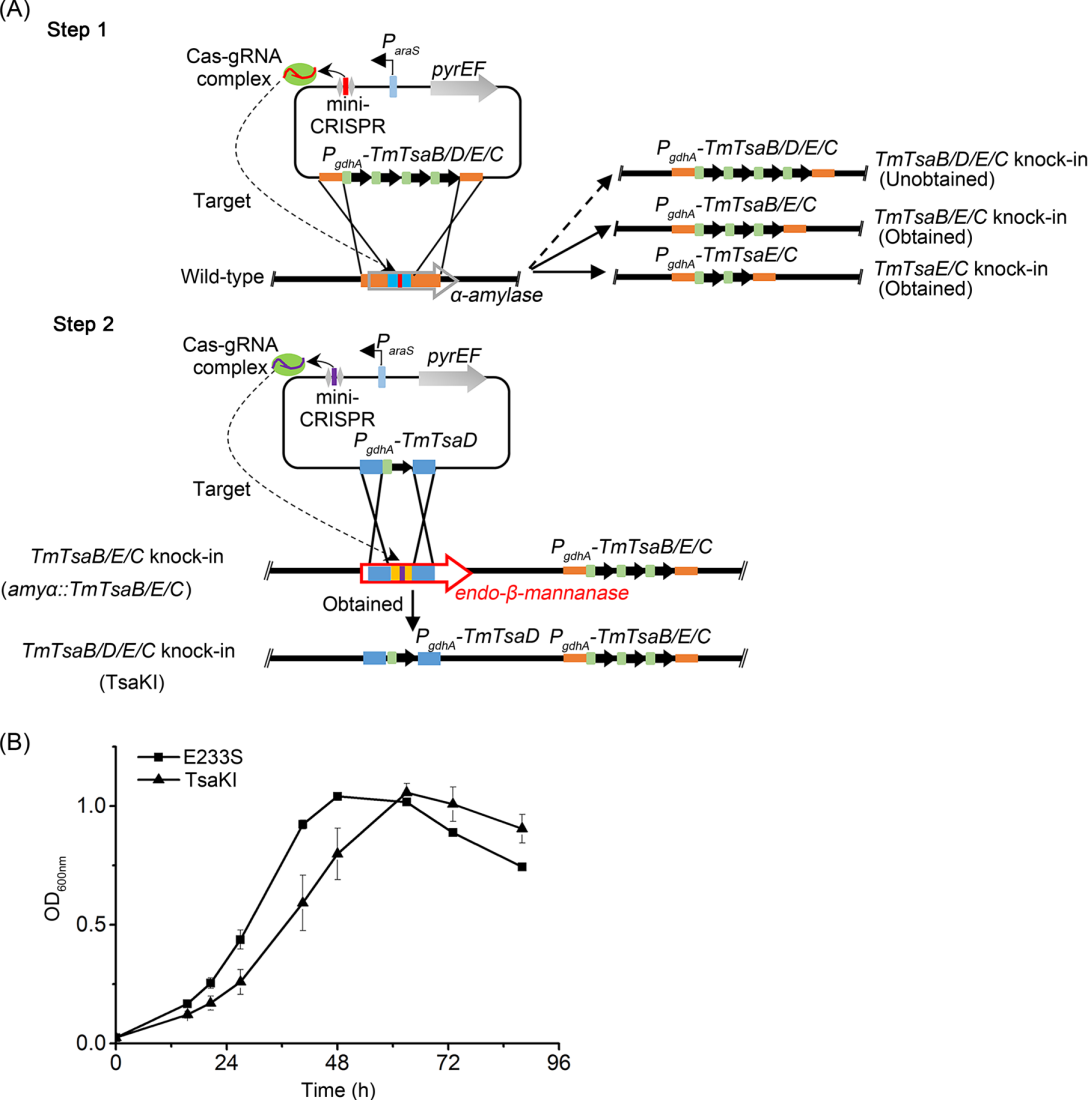
Construction of the knock‐in strain TsaKI harboring the *Thermotoga maritima* (*Tm*) t^6^A modification system. (A) Schematic for the construction of TsaKI. Step 1, plasmid was constructed to knock‐in all the four genes of the subunits of *Tm* t^6^A modification system, *TmTsaB/D/E/C*, at the *α‐amylase* locus of *Saccharolobus islandicus* genome using the endogenous CRISPR‐Cas‐based method. Strains containing three (*TmTsaB/E/C*) and two (*TmTsaE/C*) genes of the subunits were obtained. Step 2, *TmTsaD* was knocked in at the locus coding for the β‐mannanase using the obtained *TmTsaB/E/C* knock‐in strain, yielding TsaKI. Green rectangle, the promoter of *gdhA* (*P_gdhA_*). Black arrow, the genes of *TmTsaB/C/D/E*. (B) Growth curves of the wild type (E233S, filled square) and TsaKI (filled triangle). The growth curves were drawn based on averages of OD values at 600 nm of three independent cultures. Error bars indicate the standard deviation. t^6^A, N^6^‐threonylcarbamoyl adenosine.

**Figure 4:**
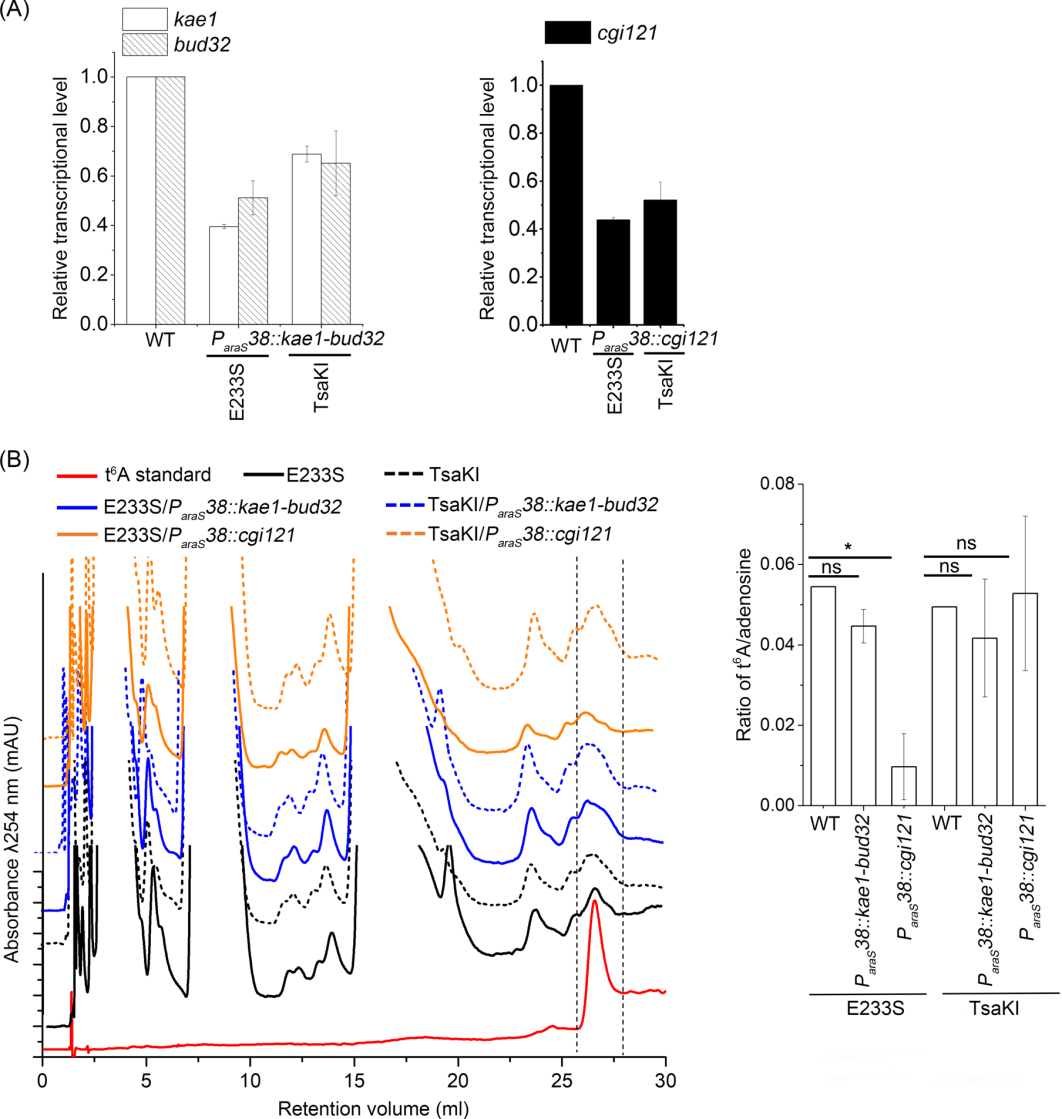
Knock‐down analysis of genes encoding *Sis*KEOPS subunits in E233S and TsaKI. (A) RT‐qPCR of *kae1‐bud32* and *cgi121* knock‐down strains with (TsaKI) and without the bacterial system (E233S). (B) Representative HPLC profiles of nucleoside composition from each sample (left) and quantification (right) showing the average t^6^A content normalized to the content of adenosine (*n* = 2 independent experiment samples, ±SD). The samples were from the wild‐type strain E233S (black), TsaKI (dashed black), *kae1‐bud32* knock‐down in E233S (blue), *cgi121* knock‐down in E233S (orange), *kae1‐bud32* knock‐down in TsaKI (dashed blue), and *cgi121* knock‐down in TsaKI (dashed orange), respectively. The t^6^A standard is shown in red. The t^6^A levels were calculated as the average ratio of t^6^A/adenosine. One‐way analysis of variance was used for the statistical analysis. *p < 0.05. ns, not significant; RT‐qPCR, real‐time‐quantitative polymerase chain reaction. HPLC, high performance liquid chromatography.

In bacteria, TsaC (Sua5 homolog in bacteria) and the TC‐transferase complex TCT complex (TsaB/TsaD/TsaE) function in t^6^A biosynthesis^36^, ^37^, ^38^. To complement the t^6^A modification role of aKEOPS in *S. islandicus*, we first tested if the bacterial t^6^A modification system could be expressed in *S. islandicus* by construction of strains overexpressing each of the genes of the t^6^A modification system from the hyperthermophilic bacteria *Thermotoga maritima* ^39^. Expression of each gene of the bacterial t^6^A modification system was verified by Western blotting using antibody against the hexa‐histidine tag (Figure S3A). Then, a knock‐in plasmid was constructed carrying *TmTsaB/TsaD/TsaE/TsaC*, a tandem array of four genes, each of which was fused to the constitutive promoter of glutamate dehydrogenase (*P*_*gdhA*_) of *S. islandicus* and expressed without the His‐tag. After transformation and colony screening, genotypes having only two and three of the genes, *amyα::TmTsaE/TsaC* and *TmTsaB/TsaE/TsaC*, respectively, were obtained, suggesting that recombination between their *P*_*gdhA*_ promoters (586 bp) could have occurred during the transformation and gene‐editing processes (Figure S3B,C). Then, the fourth gene *TmTsaD* was inserted into a gene encoding the endo‐β‐mannanase in the *amyα::TmTsaB/TsaE/TsaC* strain, yielding a strain containing the complete *T. maritima* t^6^A modification system (named as TsaKI) (Figure 3A). However, the t^6^A level in TsaKI was similar to, rather than significantly higher than, that in wild‐type E233S (Figure 4B). Considering that each subunit is able to be expressed individually, and the bacterial complex can be formed using individually expressed and purified proteins^39^, we assume that the introduced bacterial t^6^A modification system is functional in *S. islandicus*. The fact that there was no increase in the t^6^A level in TsaKI is possibly due to the fact that the t^6^A level is already at the upper limit in E233S. The growth of TsaKI was slightly slower than that of the wild‐type E233S (Figure 3B). It is not clear whether this could be due to disruption of the endo‐β‐mannanase gene now.

### The bacterial t^6^A modification system complements the t^6^A modification defect in the Cgi121 knock‐down mutant

To analyze whether aKEOPS has functions other than t^6^A modification, the KEOPS knockout plasmids were introduced into TsaKI along with E233S again. We found that similar transformation frequencies were obtained for all KEOPS knock‐out plasmids in both strains, which was much lower than that of pSeSD, a reference plasmid, suggesting that these transformants could carry escape mutations that inactivate the CRISPR immune responses. Indeed, analysis of their genotypes revealed that all transformants were wild type (data not shown), indicating that KEOPS subunits have other essential functions in vivo in addition to the t^6^A modification.

Since all KEOPS subunits are essential either in E233S or TsaKI, we performed a gene knock‐down analysis to identify the essential function in *S. islandicus*. A previous transcriptomic study showed that the transcripts of *pcc1*, *pcc1‐like*, *cgi121*, and *bud32* were quite low (FPKM∼60–140), while the transcript of *kae1* was at a moderate level (FPKM∼800–1000)^40^. In addition, CRISPR knock‐down in this archaeon is based on Type III‐B CRISPR‐Cas systems, and a prerequisite for the gene knock‐down experiment is the presence of a protospacer‐flanking sequence in target genes matching 5ʹ‐GAAAG‐3ʹ, the 5ʹ‐handle tag derived from the CRISPR repeat sequences^41^. However, such a motif is lacking in the coding sequence of *cgi121*. Therefore, we harnessed two methods for gene knock‐down: CRISPR‐Cas‐based mRNA cleavage for *pcc1*, *pcc1‐like*, *kae1*, and *bud32*, and replacement of the native promoters with mutant *P*_*araS*_ (*P*_*araS*_ *24* or *P*_*araS*_ *38*)^42^ for *cgi121* and the *kae1‐bud32* operon (Figure S4A,B). Proteins can only be slightly expressed in the presence of sucrose and induced (about 20‐fold) in the presence of arabinose with the mutant promoters, and the expression level with the *P*_*araS*_ *38* mutant is expected to be a quarter of that with *P*_*araS*_ *24* in sucrose^42^. As shown in Figure 4A, the transcriptional levels of *kae1*, *bud32*, and *cgi121* in the *P*_*araS*_ *38* replacement strains were reduced to 40%–70% of those of the wild type in E233S and TsaKI in sucrose medium.

To determine whether the t^6^A modification level was affected in the knock‐down strains, we estimated their t^6^A modification levels by high performance liquid chromatography (HPLC). Knock‐down of *kae1‐bud32* in E233S or TsaKI did not lead to any significant difference in t^6^A levels as compared with the control (Figure 4B). In contrast, the *cgi121* knock‐down led to a marked reduction (*p* = 0.029) in the t^6^A modification level in the knock‐down E233S strain. However, the t^6^A modification level in the *cgi121* knock‐down TsaKI strain did not change significantly as compared with that in E233S (Figure 4B). The mRNA level of *cgi121* knock‐down in E233S was 0.44 ± 0.01 of that in the wild type, close to the *cgi121* knock‐down ratio in TsaKI (0.52 ± 0.07) (Figure 4A). Therefore, the relatively higher t^6^A levels in TsaKI should not be due to Cgi121 knock‐down efficiency, but due to the introduction of the Tsa system. The results indicate that Cgi121 is critical for t^6^A modification in E233S. The fact that knock‐down of *cgi121* in the TsaKI strain did not affect the t^6^A modification level confirms that the bacterial t^6^A modification system complements the reduction of the t^6^A modification level of *S. islandicus* KEOPS.

### The *cgi121* knock‐down strains show growth retardance

**Figure 5:**
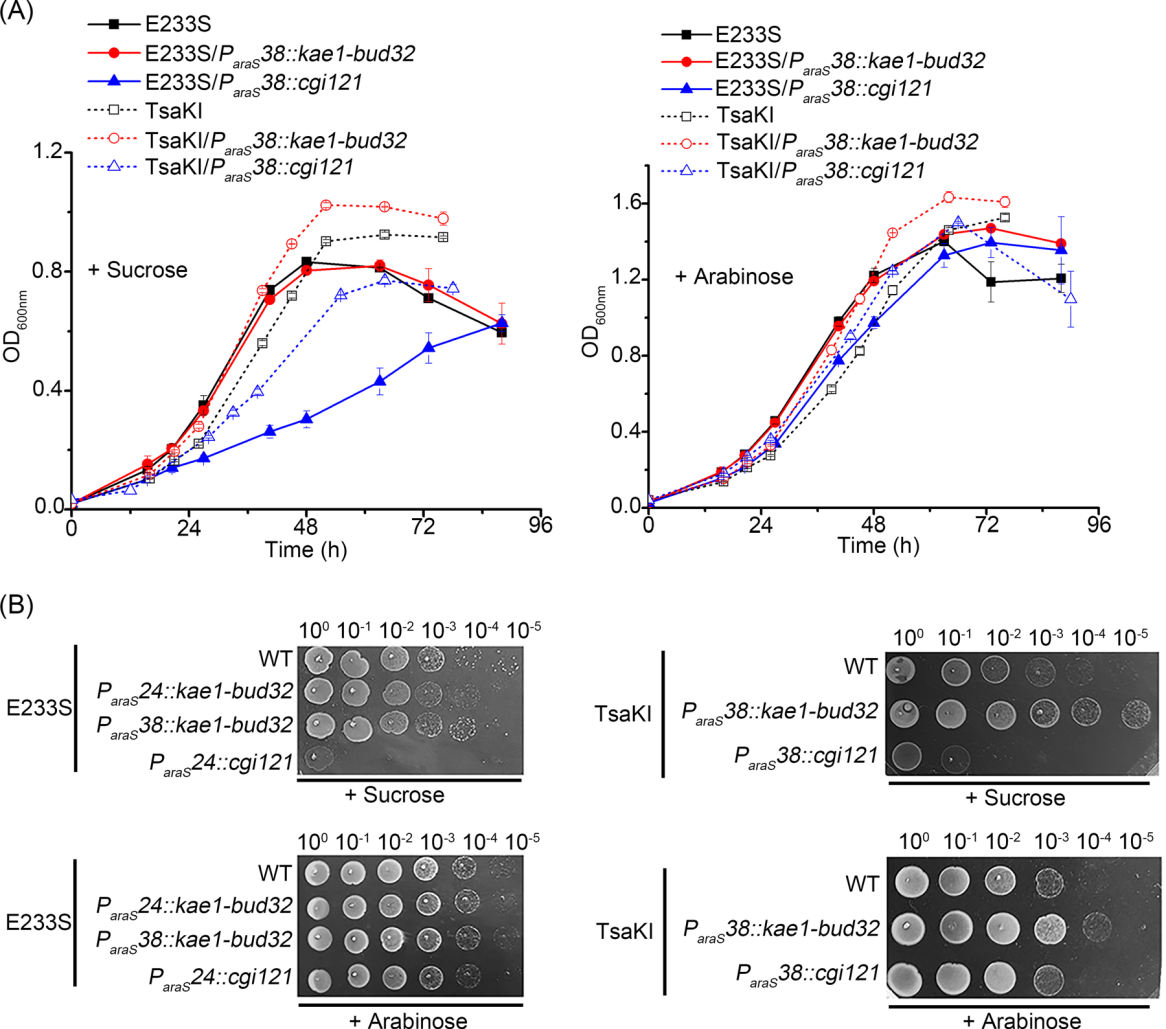
Growth analysis of *kae1‐bud32* and *cgi121* knock‐down strains in E233S and TsaKI. (A) Liquid growth. The OD values at 600 nm were measured every 6 or 12 h. The growth curves were drawn based on an average of three independent cultures. Error bars indicate standard deviations. Media containing sucrose (left) and arabinose (right) were used. (B) Spot assay using 10 μl of a series of 10‐fold dilutions prepared from a starting suspension of an OD_600_ of 0.2. The samples were spotted on the plates with media containing sucrose (above) or arabinose (bottom). The plates were cultured at 75°C for 5 days before photographing.

To reveal the additional function of aKEOPS, we analyzed the growth of the *cgi121* knock‐down and the *kae1‐bud32* knock‐down strains in liquid medium and on plates. As shown in Figure 5A, while *kae1‐bud32* knock‐down E233S did not show any difference compared with the wild‐type E233S, the *cgi121* knock‐down E233S strain showed severe growth retardation in sucrose medium. Interestingly, the *cgi121* knock‐down TasKI strain grew better than the *cgi121* knock‐down E233S, although it still grew apparently slower than E233S and TsaKI, while the *kae1‐bud32* knock‐down TsaKI grew slightly faster than TasKI. On plates, the growth differences of the strains were similar to those in the liquid (Figure 5B). For example, the wild‐type E233S was able to grow at dilutions from 10^0^ to 10^−5^, but *cgi121* knock‐down E233S only grew at 10^0^ dilution (Figure 5B). The growth defect of the *cgi121* knock‐down TsaKI was not fully rescued, consistent with the growth curves (Figure 5A,B). Intriguingly, the growth differences disappeared when the strains were cultivated in medium containing arabinose (Figure 5A). The results indicate that KEOPS has an additional function independent of the t^6^A modification level that is related to cell growth.

Although the transcriptional level of *kae1*‐*bud32* decreased in the *kae1‐bud32* knock‐down strain, there is no apparent difference in growth between the wild type and *kae1‐bud32* knock‐down strains (Figure 5). This may be due to the relatively high levels of Kae1 and Bud32 in the cells. The amount of functional KEOPS complex might be limited by the subunit with a lowest amount in the cell, which might be Cgi121, and the knock‐down of *kae1‐bud32* did not affect the formation of the functional KEOPS complex. The exact mechanism needs further investigation.

### Cgi121 shows dsDNA binding activity

In t^6^A modification, tRNA recruitment and recognition is the key step, in which Cgi121 is responsible for tRNA recruitment, while other subunits, Pcc1, Kae1, and Bud32, contribute to specific tRNA recognition^11^. Interestingly, the reconstructed *S. cerevisiae* KEOPS complex shows ssDNA and dsDNA binding activity in vitro^13^. Hence, we propose a hypothesis that there might be a similar nucleic acid binding mechanism of KEOPS functions shared in DNA repair and t^6^A modification. To reveal this, we analyzed the DNA binding activity of Cgi121 and other subunits and subcomplexes. We found that Cgi121 and Bud32 were able to bind to long dsDNA (Figure S5A), although the binding activity of Bud32 was much weaker than that of Cgi121. Intriguingly, Kae1 and Pcc1 in combination, not Kae1 or Pcc1 alone, also showed dsDNA binding activity (Figure S5B).

**Figure 6:**
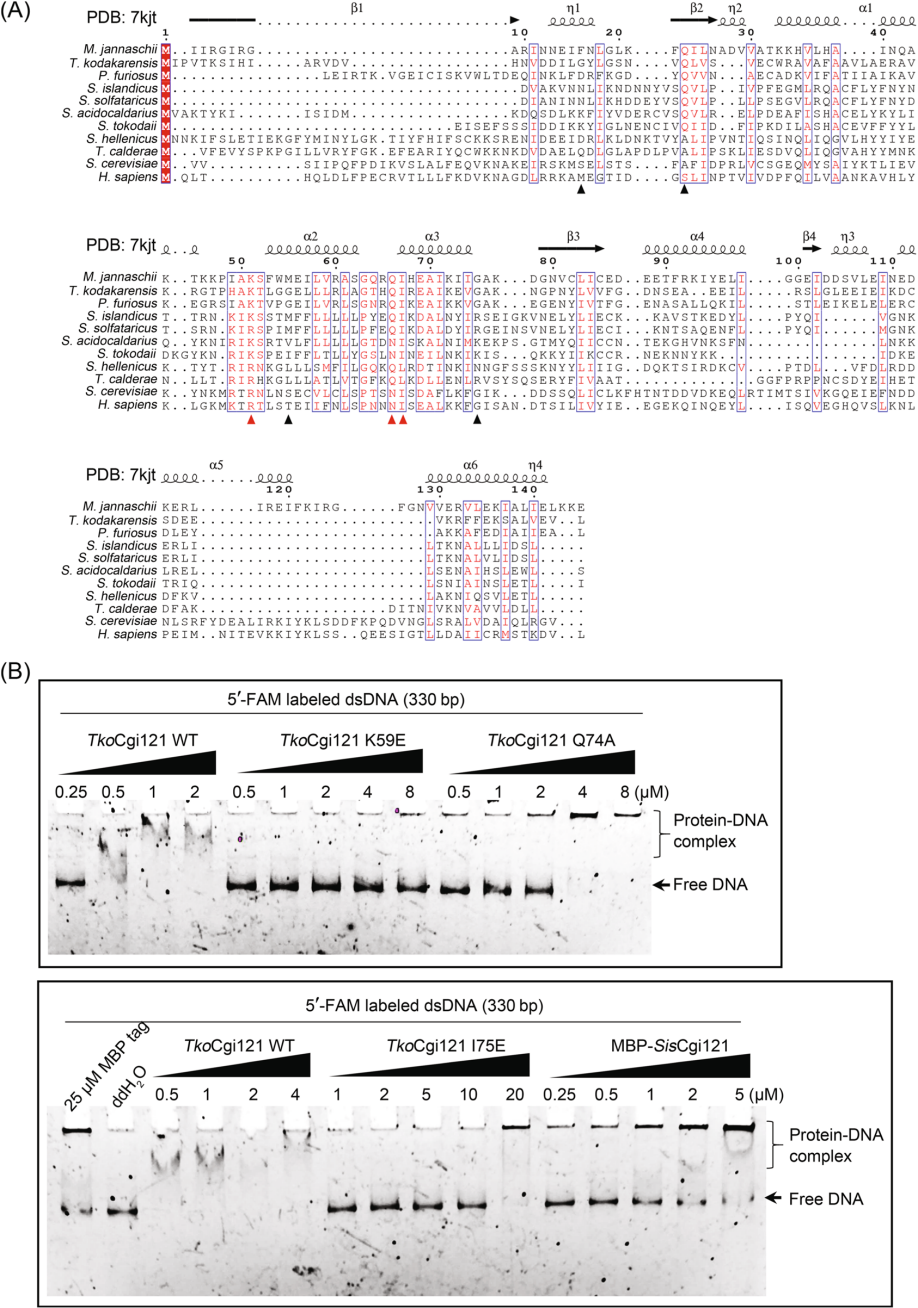
Analysis of the DNA binding ability of wild‐type Cgi121 and its mutants. (A) Structure‐based sequence alignment of Cgi121. Sequence alignment was performed on MAFFT (v7.505) and modified by Escript 3.0. The residues of the 3ʹ CCA tail ssRNA binding module of Cgi121 are shown as solid triangles and the most conserved three are colored in red triangles. (B) Binding of different Cgi121 proteins for dsDNA (330 bp random sequence). FAM‐labeled dsDNA (1 nM) was incubated with samples of the indicated concentrations of Cgi121 at 37°C for 30 min. Native PAGE gels were used for the analysis. The gels were scanned using an Amersham ImageQuant 800 after electrophoresis. The detailed reaction conditions are described in the Materials and Methods. ssRNA, single‐stranded RNA.

It was shown that *M. jannaschii* Cgi121 is an ssRNA binding protein that can specifically recognize tRNA 3ʹ CCA tail^11^. Since the protein was always degraded during protein expression and purification using a 6 × His tag, an MBP tag (40 kDa) was fused to *Sis*Cgi121 for the purification. To exclude the potential effect of the MBP tag on DNA binding ability of *Sis*Cgi121, we also purified the euryarchaeal *Thermococcus kodakarensis* Cgi121 (*Tko*Cgi121), which has about 21% sequence identity to *Mj*Cgi121 and 17% sequence identity to *Sis*Cgi121. Meanwhile, the tRNA 3ʹ CCA tail binding deficient mutants K59E, Q74A, and I75E of *Tko*Cgi121 were also constructed to explore the relationship between dsDNA binding and tRNA recruitment of Cgi121 (Figure 6A). MBP‐tagged *Sis*Cgi121 was able to bind dsDNA at a relatively low concentration, although at a high concentration (25 μM), the MBP tag also bound to dsDNA (Figure 6B). Interestingly, the wild‐type *Tko*Cgi121 was able to bind to dsDNA, while the K59E and I75E mutant lost almost all of the binding activity (Figure 6B). Notably, the Q74A mutant showed partial loss of its dsDNA affinity. A similar result was reported previously for t^6^A modification by *Mj*Cgi121 mutants^11^, suggesting that the possibility of the same mechanism of dsDNA and ssRNA binding of Cgi121. To investigate whether Cgi121 only binds the CCA motif on DNA, 5ʹ‐FAM‐labeled ssDNA and dsDNA with two CCA motifs (45 nt) were analyzed. However, all substrates could not be bound by the wild‐type*Tko*Cgi121, except for the I75E mutant and *Sis*Cgi121 at a concentration of 20 μM, which is likely nonspecific binding (Figure S5C,D). These results reveal that the tRNA binding residues of *Tko*Cgi121 also contribute to its long dsDNA binding activity, but not short ss/dsDNA binding, suggesting that the putative DNA repair function of KEOPS is related to its t^6^A modification mechanism.

### Overexpression analysis of the KEOPS subunits in *S. islandicus* implies the existence of a complex regulatory mechanism between the core subunits

**Figure 7:**
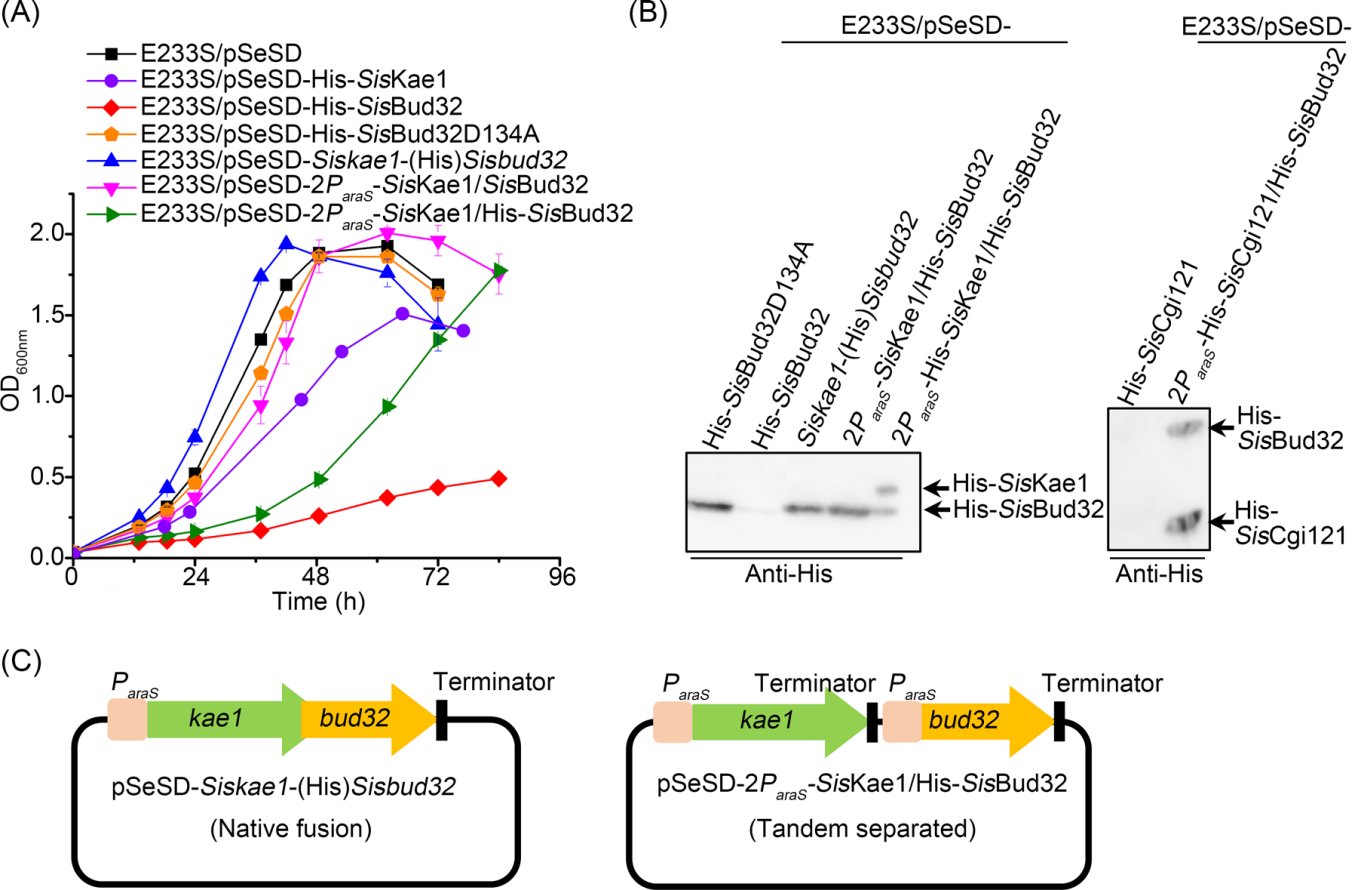
Effects of the overexpression of the KEOPS subunits on cell growth. (A) Overexpression of Kae1 and/or Bud32. The overexpression strains were cultured in arabinose medium at an initial OD_600_∼0.05. The OD values were measured every 6 or 12 h. The growth curves were drawn based on the average of three independent cultures. Error bars indicate the standard deviation. E233S/pSeSD was used as a control. (B) Western blotting analysis of the expression of Kae1, Bud32, and Cgi121. The cells were collected at the exponential phase and lysed by boiling for 10 min. The protein levels were detected using the anti‐His antibody. (C) Schematic of the constructs for *Sis*Kae1 and *Sis*Bud32 co‐expression. *kae1* and *bud32* have a 40 bp overlap in the Native fusion plasmid as in the genome. In the Tandem separated plasmid, *kae1* and *bud32* expression was controlled by two independent *araS* promoters.

To further investigate the role of KEOPS subunits in DNA repair and to gain insight into the involved mechanisms, the subunits were overexpressed alone or in different combinations in *S. islandicus*. We found that overexpression of either Kae1 or Bud32, the two core components of the KEOPS complex, inhibited cell growth, with the latter showing more profound growth inhibition than the former (Figure 7A). Interestingly, overexpression of a kinase/ATPase inactive mutant Bud32D134A did not show this inhibition, suggesting that the growth retardation was due to its kinase/ATPase activity (Figure 7A). When Kae1 and Bud32 were co‐overexpressed in a native gene organization manner (Figure 7C), no growth retardation was observed. However, cell growth was inhibited if the expression of the two genes was controlled by two separate *P*_*araS*_ promoters and induced by arabinose (Figure 7A,C). In a previous study, it was shown that the transcript of *bud32* was several folds lower than that of *kae1* ^40^. This could be attributed to the difference in the stability of the two transcripts. Alternatively, an unknown mechanism could be responsible for maintaining the ratio of Kae1 and Bud32 in vivo. Additionally, we analyzed the effect of the expression of Pcc1, Pcc1‐like, and Cgi121, and co‐expression of Cgi121 and Bud32 on the cell growth. When overexpressed alone in *S. islandicus*, the proteins of Pcc1, Pcc1‐like, and Cgi121 could not be detected by Western blotting. However, when Cgi121 and Bud32 were co‐expressed, both proteins were clearly detected (Figure 7B), indicating that Cgi121 and Bud32 may stabilize with each other in vivo. Interestingly, the growth of Cgi121 and Bud32 co‐overexpression strain showed no difference from the strain carrying the control plasmid pSeSD. Taken together, our results imply that Kae1 and Bud32 and the kinase/ATPase activity of Bud32 have significant physiological functions and that Kae1 and Cgi121 play regulatory roles in the activity of Bud32. The results also suggest that the levels of KEOPS subunits, especially Bud32, Kae1, and Cgi121, must be maintained in an appropriate proportion in *S. islandicus* for their stability and proper functioning.

## DISCUSSION

About 60 proteins are conserved in all cellular life; these include Sua5/TsaC and Kae1/TsaD/Qri7, the key enzymes in t^6^A modification^10^, ^41^. Kae1, in cooperation with Pcc1, Bud32, Cgi121, and eukaryotes' specific fifth subunit Gon7, forms the KEOPS complex to catalyze t^6^A modification^35^. Besides t^6^A modification, the KEOPS complex also plays roles in HR repair and telomere maintenance^13^, ^21^. Although it was shown that aKEOPS plays a role in t^6^A modification, it is unclear whether it has the fifth subunit and whether it has a function other than t^6^A modification. Here, we show that the paralog of Pcc1, Pcc1‐like is the fifth subunit of KEOPS in archaea and the aKEOPS complex plays an essential role in other cellular processes besides t^6^A modification, presumably DNA homologous recombinational repair.

Although archaeal Pcc1, Kae1, and Bud32 were the minimal components for t^6^A modification activity in vitro, the eukaryotic fifth subunit Gon7 deletion strain also caused severe t^6^A modification defect in *S. cerevisiae*, indicating the critical role of the fifth subunit^12^, ^35^. Therefore, we searched for the distribution of KEOPS subunits in archaea and identified a Pcc1 paralog, Pcc1‐like, that is likely the functional homolog of Gon7. In vivo genetic analysis showed that both *pcc1* and *pcc1‐like* cannot be deleted, suggesting a nonredundant role of *pcc1‐like*. In vivo and in vitro interaction analyses show that Pcc1‐like interacts with both Pcc1 and Kae1. Pcc1 inhibits the dimerization of Kae1‐Pcc1 and stabilizes the subcomplex in vitro, just like Gon7^4^, ^9^. Additionally, bioinformatics analysis shows that the Pcc1‐Pcc1‐like heterodimer interface resembles that of the eukaryotic Pcc1‐Gon7 heterodimer. Interestingly, through sequence alignment and phylogenetic analysis, we found that the evolution rate of Pcc1 and Pcc1‐like was higher than that of other KEOPS subunits and Pcc1‐like lineage seems to have a closer relationship with the Gon7 lineage than archaeal and eukaryotic Pcc1 in the phylogenetic tree (Figure 1B), which confirms that Pcc1‐like is a functional homolog of Gon7. The fifth subunit Gon7 also plays a role in t^6^A modification and DNA repair in *S. cerevisiae* and *Homo sapiens* ^5^, ^9^, ^13^, ^35^. However, because its structure is partially disordered, the mechanism of Gon7 in these functions is still unknown^4^, ^9^. Structurally, Gon7 interacts with Pcc1 and stabilizes KEOPS, forming a 1:1:1:1:1 complex^4^, ^9^. The precise role of Pcc1‐like/Gon7 in HR repair and t^6^A modification needs to be studied further. To this end, Pcc1‐like and aKEOPS as the whole complex from thermophilic archaea could be used as excellent structural and functional models for KEOPS study, taking advantage of their stability.

We demonstrate that KEOPS has a t^6^A modification level‐independent function that is essential in *S. islandicus*, a thermophilic crenarchaeon. To circumvent the possible essentiality of aKEOPS, we introduced a thermophilic bacteria‐derived t^6^A modification system^34^ into *S. islandicus* and performed genetic analysis of the KEOPS. We show that the bacterial t^6^A modification system is active in *S. islandicus* and can complement the t^6^A modification function of aKEOPS. Using this constructed knock‐in strain TsaKI, we demonstrate that all the KEOPS subunits cannot be deleted and knock‐down of Cgi121 leads to growth retardance. These results indicate that archaeal Cgi121 plays an important role in the cell besides t^6^A modification.

**Figure 8:**
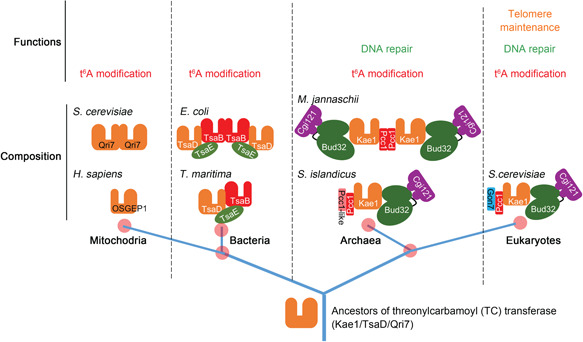
A proposed model for the evolution of threonylcarbamoyl (TC)‐transferases and its complexes. The ancestral TC‐transferase could be Kae1/TsaD, from which mitochondrial TC‐transferase and bacterial TsaD induced TC transfer. Later, the ATPase Bud32/TsaE and the dimerization subunit Pcc1/TsaB joined to form the complexes comprising minimum components and essential for t^6^A modification. Meanwhile, Cgi121 joined into the initial complex of the archaea–eukaryote linkage to recruit tRNA. In the later stage, Pcc1‐like might appear via a gene duplicate to stabilize the complex. Perhaps, since Pcc1‐like worked as a regulator in t^6^A modification, it showed the highest mutation rate among the subunits and evolved to Gon7 in eukaryotes. As Cgi121 and Pcc1‐like (Gon7) were integrated into the complex, KEOPS gained additional functions other than t^6^A modification, such as DNA repair (HR repair), before the separation of archaea and eukaryotes. In eukaryotes, KEOPS also participates in telomere maintenance, which requires DNA recombination processes to be completed by multiple HR repair proteins. HR, homologous recombination.

The HR repair pathway is one of the most complicated pathways and its mechanism has not been fully elucidated. Among the DNA repair pathways of thermophilic archaea such as *Sulfolobales* and *Thermococales*, only the HR pathway is essential and most genes involved in HR cannot be knocked out^25^, ^26^, ^33^, ^43^. Considering this, in *S. islandicus*, the pathway in which KEOPS is involved in is most likely HR repair. In halophilic euryarchaeon *H. volcanii*, *kae1*, *bud32*, and *cgi121* are essential for cell viability, whereas deletion of *pcc1* resulted in cells with higher nucleic acid content^28^. The authors did not explain the cause of this observation, but we now assume that this could be due to the presence of higher genome copy number, which may indicate that KEOPS is involved in DNA repair. *H. volcanii* is highly polyploid, with approximately 20 copies of genomes per cell, and is capable of origin‐free replication depending on DNA recombination^44^, ^45^ Therefore, in addition to t^6^A modification, *H. volcanii* KEOPS might also contribute to this polyploidy species‐specific, HR‐dependent replication mode. In humans, knock‐down of genes encoding KEOPS subunits induces DNA damage response signaling and subsequent apoptosis^8^. Therefore, involvement in DSB resection could be a conserved and ancestral function of KEOPS (Figure 8).

We found that overexpression of different subunits, their mutant, or coexpression of combinations of subunits had distinct effects on cell growth. The phenotypes resemble those in the yeast to some extent. It was reported that the activity residues of the kinase Bud32 play a critical role in HR repair in *S. cerevisiae*, and the kinase activity of *S. cerevisiae* Bud32 was inhibited by Kae1 and activated by Cgi121 in vitro^3^, ^13^, ^46^. These results suggest that there is a sophisticated regulatory mechanism of the KEOPS complex. The mechanism by which aKEOPS participates in cellular processes such as HR and how this function is coordinated with the t^6^A modification will be an intriguing subject for further investigation.

A mystery of KEOPS is the molecular basis to explain why KEOPS has three such seemingly unrelated functions, t^6^A modification, HR repair, and telomere maintenance in yeast. To date, mechanisms have been elucidated for the t^6^A modification of KEOPS, except for the precise roles of Gon7 and Pcc1 in the process. Cgi121 was reported to be a tRNA 3ʹ CCA tail and ssRNA binding protein for the recruitment of tRNA to KEOPS during t^6^A modification^11^. Interestingly, telomeres in most eukaryotes are composed of TT[A/T]GGG repeats, whose antisense strands and corresponding telomerase RNAs are CCC[T/A]AA repeats^47^. Therefore, the nucleic acids CCA binding ability of Cgi121 may be a link among the three functions of eukaryotic KEOPS. Our study revealed that Cgi121 is the only subunit that binds to long dsDNA individually, but not short ss/dsDNA (which might be not able to form a higher structure) containing a CCA motif. Strikingly, mutation at the tRNA binding residues of *Tko*Cgi121 led to a defect in long dsDNA binding ability, suggesting that the mechanisms of KEOPS in t^6^A modification and DNA repair are related^11^. The evolutionary transition from circular to linear chromosomes, which harbor telomeric structures, leads to more challenges to genome stability in eukaryotic cells. This could be the reason why multiple DNA repair proteins, especially classical HR proteins and KEOPS, also participate in telomere maintenance.

Finally, based on studies on the composition and functions of the t^6^A modification systems, we proposed a model for the evolution of the KEOPS complex and other TC‐transferases or their complexes (Figure 7). The ancestral TC‐transferase could be a single‐protein Kae1/TsaD, from which mitochondrial TC‐transferase Qri7 and bacterial TsaD originated independently^35^, ^39^. Then, ATPase Bud32/TsaE and the dimerization module Pcc1/TsaB join to form an initial KEOPS complex, which contains the minimum components and is essential for t^6^A modification^34^, ^35^. Meanwhile, Cgi121 enter the initial complex of the archaea–eukaryote lineage to recruit tRNA^11^. At a later stage, Pcc1‐like appears via gene duplication, stabilizes the complex, and improves the efficiency of t^6^A modification (Figure S6)^9^. Finally, due to the accessory role of Pcc1‐like in t^6^A modification, Pcc1‐like shows the highest mutation rates among the subunits and evolves into Gon7 in eukaryotes. During the later stages, as the additional subunits join, KEOPS gains functions other than t^6^A modification, such as HR repair, before the separation of archaea and eukaryotes. In eukaryotes, KEOPS is also involved in telomere maintenance, which also needs a process of DNA recombination, like other HR repair proteins such as Mre11 and Rad50^48^, ^49^.

## MATERIALS AND METHODS

### Strains and growth conditions

*S. islandicus* strain REY15A (E233S) (Δ*pyrEF*Δ*lacS*) (hereafter E233S) and its transformants were cultured as described previously^50^ . d‐arabinose (0.2% w/v) was used for cell growth to induce protein overexpression controlled by different *araS* promoters.

### Construction of plasmids and strains

The strains and vectors used and constructed in this study are listed in Tables S1 and S2. Oligonucleotides for spacers, *P*_*araS*_ mutant promoters, fluorescence‐labeled ssDNA, and primers for PCR and real‐time‐quantitative PCR (RT‐qPCR) are listed in Table S3. For the construction of *T. maritima* t^6^A biosynthesis system knock‐in strain TsaKI, constitutive expression vector pSeGP was constructed by replacing the *araS*‐SD sequence with the promoter of *S. islandicus* glutamate dehydrogenase (*P*_*gdhA*_) at the site between *Sph*I and *Nde*I of pSeSD^51^. The gene for the TsaB, TsaC, TsaD, or TsaE was inserted into the *Nde*I and *Sal*I site of pSeGP individually. Then, the cassettes for the TsaB, TsaC, TsaD, and TsaE were amplified and ligated into pRSF‐Duet1 sequentially in the order of TsaB‐TsaD‐TsaE‐TsaC. The homologous arms (L‐arm and R‐arm) of *amyα* (SiRe_1098) were ligated via gene splicing by overlap extension PCR with *Kpn*I/*Xho*I sites in between and inserted into the *Sph*I/*Bln*I site of pUC19. Next, the cassette of *Tm*TsaB‐TsaD‐TsaE‐TsaC was inserted into the *Kpn*I/*Xho*I site between the two homologous arms. Finally, the fragment containing the homologous arms and *Tm*TsaB‐TsaD‐TsaE‐TsaC was amplified and inserted into the *Sph*I/*Bln*I site of the pGE vector^32^, yielding a vector pGE‐*Tm* *TsaB/D/E/C* *‐*KI. To construct pGE‐*Tm* *TsaD* *‐*KI, the spacer was designed to target the endo‐β‐mannanase (SiRe_2238) and the fragment containing the homologous arms and *Tm* *TsaD* was inserted into the *Sph*I/*Bln*I site of pGE. For construction of the vectors for the gene knock‐down, the corresponding spacers were inserted into the mini‐CRISPR locus in pGE^32^. For the construction of the knock‐out strain, the corresponding spacers were inserted into the mini‐CRISPR locus and the fragment containing the homologous arms was inserted into the *Sph*I/*Xho*I site of pGE. To construct vectors for protein overexpression in *S. islandicus*, the gene fragments were amplified by PCR, digested with restriction enzymes, and inserted into the *Nde*I and *Sal*I site of pSeSD^52^. To construct vectors for protein co‐overexpression in *S. islandicus*, another multiple clone site (*Xho*I/*Nhe*I) with an *araS* promoter was introduced into pSeSD, yielding pSeSD‐2P_araS_ for plasmid construction.

To construct the knock‐in strain TsaKI harboring *T. maritima* t^6^A biosynthesis system, the vector pGE‐*TmTsaB*/*D*/*E*/*C*‐KI was transformed into E233S. The transformants were screened on uracil‐free media and verified by PCR using flanking primers (Table S3). The vector pGE‐*TmTsaD*‐KI was transformed into *amyα::TmTsaB/TsaE/TsaC*, yielding the strain TsaKI containing the whole *T. maritima* t^6^A biosynthesis system. The vectors for knockdown, knockout, and overexpression of KEOPS genes were transformed into E233S to obtain the corresponding strains.

### Bioinformatic analysis

BLAST and genome context analysis was performed to identify Sua5 and KEOPS homolog sequences in archaea and eukaryotes^53^. The evolutionary distances were calculated using the *p*‐distance method^54^. For phylogenetic analysis of Pcc1 and Gon7, 126 homolog sequences from 82 representative species of each phylum of archaea and eukaryotes were aligned by MAFFT v7^55^, ^56^. The evolutionary history was inferred using the maximum likelihood method based on the Le_Gascuel_2008 model^57^. The phylogenetic tree was constructed with MEGA7 and drawn to scale, with branch lengths measured in the number of substitutions per site. All positions with site coverage ≤95% were eliminated. Evolutionary analyses were conducted in MEGA7 and prettified by iTOL (https://itol.embl.de/)^58^.

### Protein expression and purification

For protein expression, the vectors were transformed into the *E. coli* BL21(DE3)‐Codon plus‐RIL strain. No‐tagged or hexa‐histidine (6 × His)‐tagged *Sis*Kae1 (Kae1 from *S. islandicus*) and *Sis*Bud32 were expressed using pET15b. His‐tagged *Sis*Pcc1‐like, *Tko*Cgi121, and *Tko*Cgi121I75E were expressed using pRSF‐Duet1. Maltose‐binding protein (MBP)‐ and His‐tagged *Sis*Cgi121 and *Sis*Pcc1 were expressed using pMALc2X. No‐tagged *Sis*Kae1 were co‐expressed with His‐tagged *Sis*Pcc1 using pRSF‐Duet1. His‐tagged *Sis*Pcc1‐like was coexpressed with Flag‐tagged *Sis*Kae1 and *Sis*Pcc1 using pRSF‐Duet1, respectively. The expression was induced by the addition of 0.1 mM IPTG to 1.0 l of LB media at OD_600_ 0.4–0.6 and the cells were cultivated at 37°C for 4 h. The proteins were purified using nickel affinity chromatography (Ni‐NTA) coupled to anion exchange (HiTrap Q FF) and SEC (Superdex 200 Increase 10/300) in a buffer containing 50 mM Tris‐HCl pH 8.0, 5% glycerol, and 50–500 mM NaCl depending on different proteins.

For protein expression and purification in *S. islandicus*, strains carrying the constructed pSeSD‐based vectors were cultivated in sucrose medium. Expression was induced by the addition of 0.2% d‐arabinose to 1.0 l cultures at an OD_600_ of 0.2 and the cells were cultured at 75°C for 24 h. His‐tagged *Sis*Pcc1 and *Sis*Kae1 were expressed separately. His‐tagged *Sis*Bud32 and no tagged *Sis*Kae1 were coexpressed by cloning the *kae1‐bud32* operon into pSeSD. The subsequent protein purification was the same as described above.

### Pull‐down assay

His‐tag pull‐down was performed using 10 μM His‐*Sis*Pcc1‐like protein mixed with 12 μM no‐tagged *Sis*Kae1, or 10 μM His‐*Sis*Kae1 mixed with 12 μM no‐tagged *Sis*Bud32, in 200 μl binding buffer (50 mM NaCl, 50 mM Tris‐HCl pH 8.0). The mixture was incubated with 50 μl of pre‐equilibrated Ni‐NTA nickel‐charged affinity resin. Binding was allowed to proceed for 10 min at 4°C with mild shaking, followed by washing three times with the wash buffer (binding buffer containing 10 mM imidazole). The resin was resuspended in the elute buffer (binding buffer containing 400 mM imidazole) and the supernatant was analyzed by 15% SDS‐PAGE and Coomassie blue staining.

MBP‐tag pull‐down was conducted using 10 μM MBP‐*Sis*Cgi121‐His mixed with 12 μM His*‐Sis*Bud32 in 200 μl of binding buffer. The mixture was incubated with 50 μl of pre‐equilibrated amylose resin. Binding was allowed to proceed for 10 min at 4°C with mild shaking, followed by washing in binding buffer three times. The resin was resuspended in elute buffer (binding buffer containing 10 mM maltose) and the supernatant was analyzed by 15% SDS‐PAGE and Western blotting.

### SEC analysis

SEC analysis of single proteins or combinations of the subunits was conducted using the AKTA basic 10 FPLC system (GE healthcare) and a Superdex 200 Increase 10/300 SEC column. Chromatography was performed in a running buffer (200 mM NaCl, 50 mM Tris‐HCl pH 8.0, and 5% glycerol). For complex reconstitution, 30 μM *Sis*Pcc1‐like was mixed with 20 μM *Sis*Kae1/*Sis*Pcc1‐like, or 6 μM *Sis*Pcc1‐like mixed with 30 μM *Sis*Kae1, in 600 μl of running buffer at 60°C for 30 min before loading onto the column.

### Growth curve and dot assay

The growth curves of the *S. islandicus* strains were measured as follows: each strain was precultured in a flask containing 25 ml of medium at 75°C for 24 h until the culture reached an OD_600_ of 0.4–0.6. Cells were collected and resuspended in 25 ml of ddH_2_O and then inoculated into 30 ml of medium to an estimated OD_600_ of 0.04 and cultured at 75°C in a 100 ml flask with shaking. The cell densities were measured at the indicated time intervals. Each strain was analyzed in duplicate, and the measurements were performed three times independently. For the growth observation on plates, an overnight‐grown culture was diluted to an estimated OD_600_ of 0.2. Cells were diluted in 10‐fold gradience and spotted on plates. Plates were incubated at 75°C for 5 days before photos were taken.

### Analysis of t^6^A modification and transcription by RT‐qPCR

Bulk tRNA were isolated according to the reported method^59^ using hot acid‐saturated phenol, with slight modifications. In particular, the cells were grown in 25 ml of medium at 75°C to OD_600_ of 0.8 before collection. The isolated bulk tRNA was analyzed according to the reported protocol^60^. Briefly, tRNA (5 μg) was boiled for 2 min and cooled down on ice immediately. Then, the tRNA was digested with 10 U Nuclease P1 (New England Biolabs) at 37°C overnight, followed by dephosphorylation using 10 U bacteria alkaline phosphatase (ThermoFisher Scientific) at 65°C for 2 h. The ribonucleotides were analyzed using an Ultimate 3000 HPLC (ThermoFisher Scientific) using a Zorbax SB‐C18 (250 × 4.6 mm, 5 μM) reverse‐phase column (Agilent). Ribonucleotides were separated by linear gradient elution from 2% to 12.5% buffer B at a flow rate of 1.5 ml/min. The compositions of the HPLC buffers were as follows: (A) 250 mM NH_4_Ac, pH 6.5; (B) 40% acetonitrile. The peaks were analyzed using the software Chromeleon 7, and the relative t^6^A modification level was described as the ratio t^6^A/adenosine.

For RT‐qPCR analysis, when the cultures were grown to an OD_600_ of 0.8, cells of 2 ml cultures were collected by centrifugation. Total RNA was isolated with 1 ml of Trizol (TransGene Biotech), followed by chloroform and isopropanol extraction. The RNA was precipitated by 75% ethanol and dissolved in 50 μl of DEPC water. RNA (500 ng) was used for cDNA synthesis using an Evo M‐ML kit (Accurate Biology) according to the manufacturer's instructions. RT‐qPCR was conducted using the CFX96 Real Time PCR detection system (Bio‐Rad) using a SYBR Green Pro Taq HS kit (Accurate Biology) according to the manufacturer's instructions.

### Electrophoretic mobility shift assay (EMSA)

The EMSA assay was performed in a 20 μl reaction mixture containing 10 nM fluorescence‐labeled probes (Table S3) and the indicated proteins (0–20 μM) in a binding buffer (50 mM Tris‐HCl pH 8.0, 100 μM ATP). The reactions were incubated at 37°C for 30 min. The products were separated by 8% PAGE in 0.5 × TBE at 4°C. The gels were imaged using Amersham ImageQuant 800 (Cytiva).

## AUTHOR CONTRIBUTIONS

Pengju Wu, Qihong Huang, and Yulong Shen designed the research. Qihong Huang and Yulong Shen supervised the project. Pengju Wu, Qihong Huang, and Qi Gan performed the experiments. Xuemei Zhang, Yunfeng Yang, and Yuanxi Xiao helped with the data analyses. Pengju Wu and Qihong Huang wrote the manuscript. Qunxin She, Jinfeng Ni, Qihong Huang, and Yulong Shen revised the manuscript.

## ETHICS STATEMENT

This article does not contain any studies with human participants or animals performed by any of the authors.

## CONFLICT OF INTERESTS

The authors declare no conflict of interests.

## Supporting information

Supporting information.

## Data Availability

All data supporting the findings of this study are available within the article and the Supporting Information, or from the corresponding author upon reasonable request.
