# Supplementary material for: The archaeal KEOPS complex possesses a functional Gon7 homolog and has an essential function independent of the cellular t6A modification level
Source: mLife. 2023 Jan 8;2(1):11–27. doi: 10.1002/mlf2.12051 (PMC10989989; doi:10.1002/mlf2.12051)
Supplement: Supplementary file 1 — Supporting information. [file MLF2-2-11-s001.pdf]

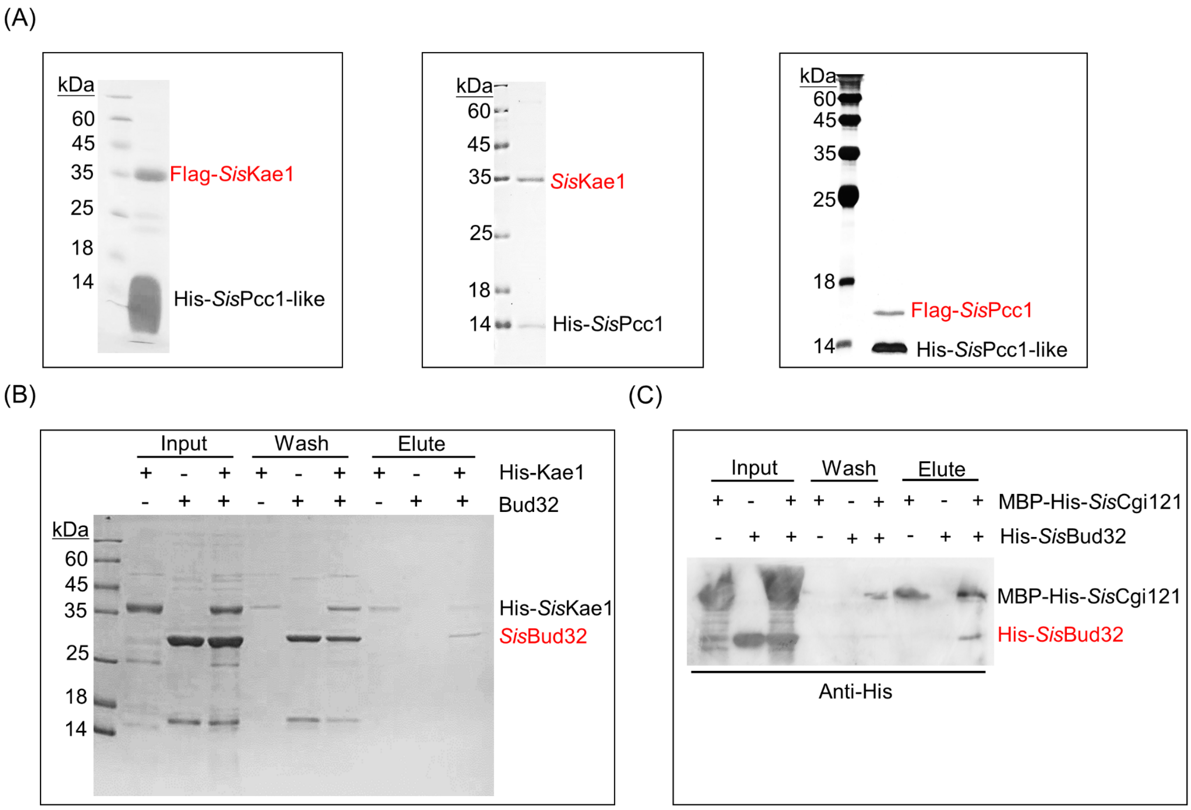

Figure S1. Pair-wise interaction analysis of aKEOPS subunits by pull down. (**A**) Assay using proteins expressed and purified from *E. coli*. The interactions between *Sis*Kae1 and *Sis*Pcc1-like, *Sis*Kae1 and *Sis*Pcc1, and *Sis*Pcc1 and *Sis*Pcc1-like were verified by the ability to pull down the target proteins (red) with the His-tagged proteins co-expressed. The final eluted samples from Ni-NTA beads were analyzed by SDS-PAGE. (**B**) *In vitro* pull down assay using purified His-*Sis*Kae1 as a bait and non-tagged *Sis*Bud32 as a prey. The samples were analyzed by SDS-PAGE and Coomassie blue staining. (**C**) *In vitro* pull down assay using MBP-His-*Sis*Cgi121 as a bait and His-*Sis*Bud32 with amylose resin. The samples were analyzed by Western blotting. In (B) and (C), all the proteins were expressed in *E. coli* individually and purified by affinity chromatography. The asterisk indicates a non-specific band in the MBP-His-SisCgi121 sample that cannot bind to amylose resin.

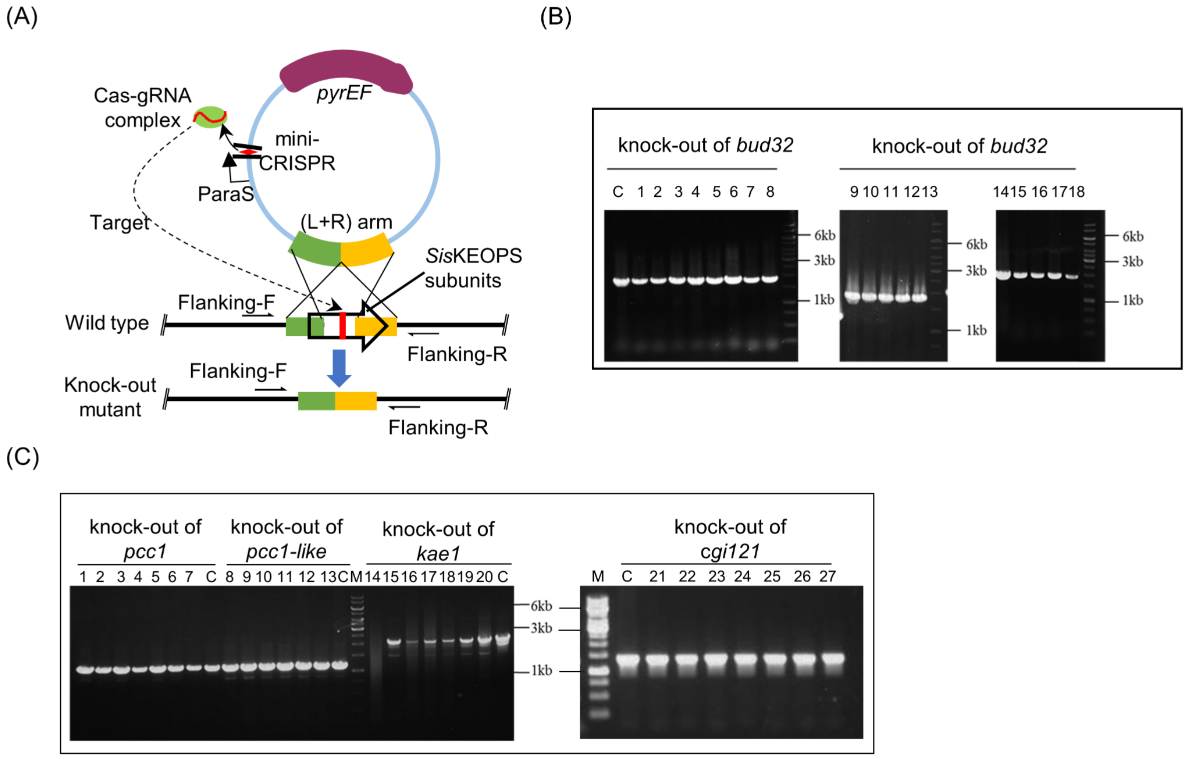

Figure S2. PCR analysis of the colonies obtained in the *Sis*KEOPS gene knock-out experiments. (A) Schematic of the gene knock-out strategy. **(B)** Screening for *bud32* knock-out colonies. **(C)** Screening for *pcc1*, *pcc1-like, kae1* and *cgi121*knock-out colonies. M, molecular size marker. C, control (E233S). The numbers indicate colonies picked from the plates after transformation.

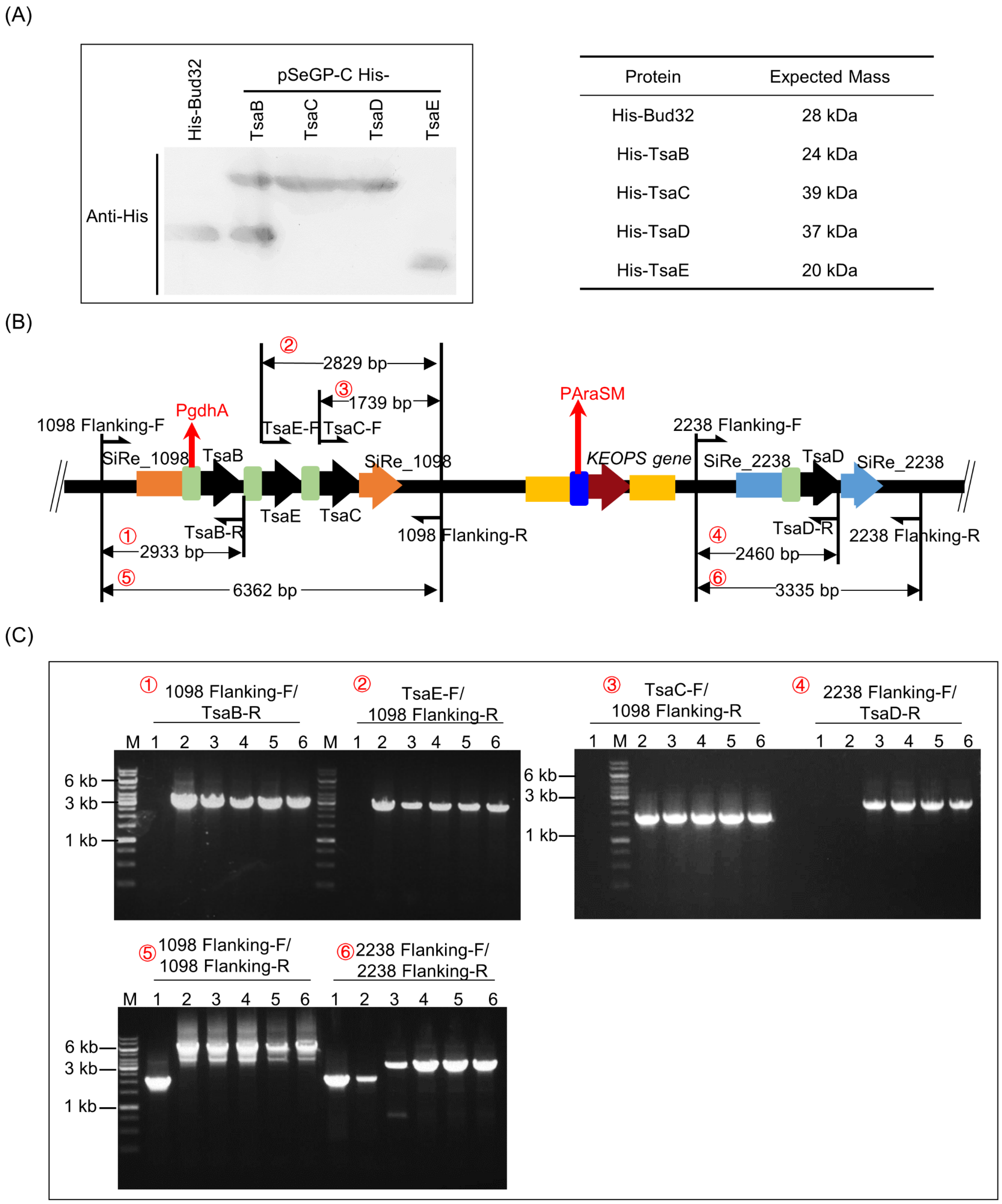

Figure S3. Confirmation of protein expression of TmTsaB/C/D/E in the overexpression strains of E233S and verification of the insertion of *TmTsaB/C/D/E* in TsaKI. (A) Western blotting analysis of strains over expressing each of TmTsaB/TsaC/TsaD/TsaE with C terminal His-tag (left) using the shuttle vector pSeGP. About 2.5×10^{8} cells were collected. Purified His-tagged Bud32 was used as a positive control. Expected molecular mass of each protein is shown on right. (**B**) Schematic for the primers at the knock-in locus and the expected PCR products. The numbers in red indicated PCR products of their corresponding numbers in (B). (**C**) Analysis of the PCR products using primers at the *α-amylose* (*SiRe_1098*) and the *β-mannanase* (*SiRe_2238*) loci. Lanes 1, E233S; 2, E233S/*TmTsaBEC::amyα*; 3, TsaKI; 4, TsaKI/*P_{araS}24::kae1-bud32.*; 5, TsaKI/*P_{araS}38::kae1-bud32*. 6, TsaKI/*P_{araS}38::cgi121*.

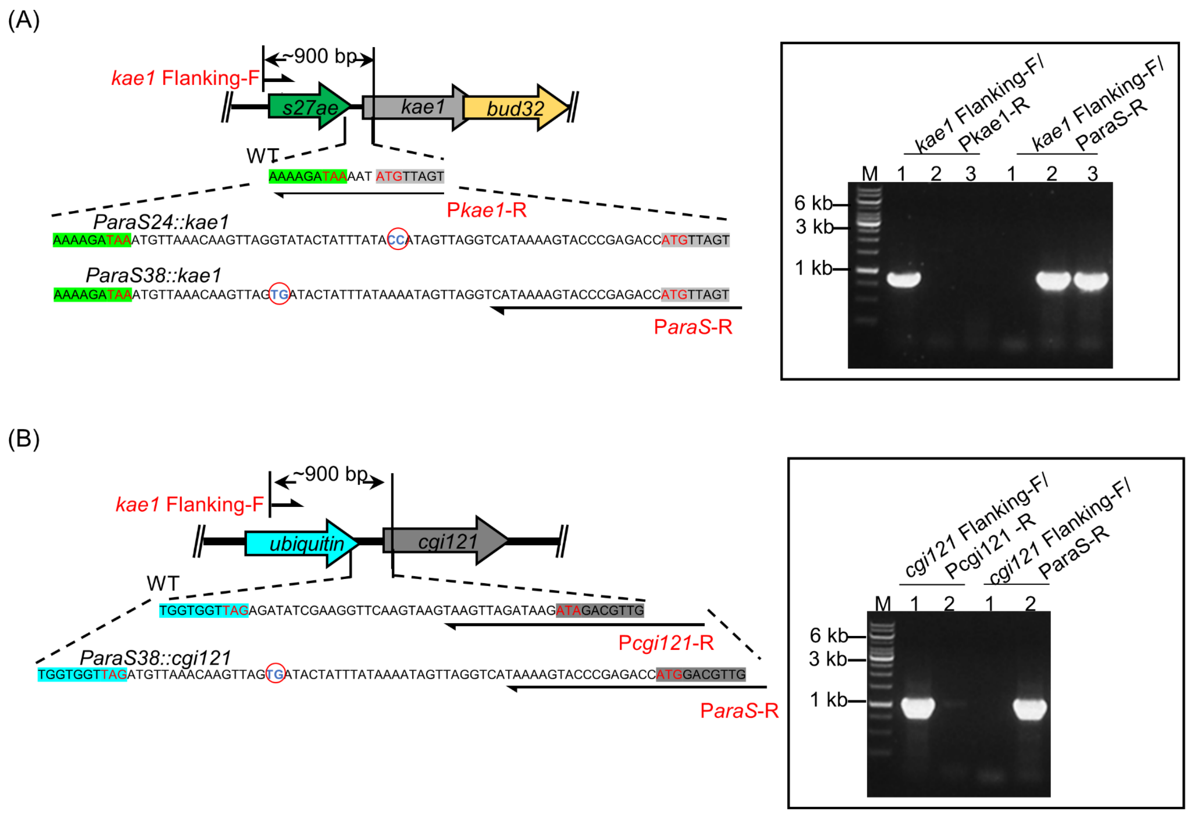

Figure S4. PCR verification of the promoter replacement of the KEOPS genes in TsaKI. (**A**) Analysis of PCR products using primers at the loci of *kae1* promoters (right). The schematic for the promoter replacement of *kae1-bud32* is shown on the left. The start and stop codons of *kae1* (or *cgi121* in D) and its upstream gene are indicated in red. The two nucleotides in red circular are mutations of the wild type arabinose promoters. 1, TsaKI; 2, TsaKI/P_{araS}*24::kae1-bud32*.; 3, TsaKI/P_{araS}*38::kae1-bud32*. (**B**) Analysis of the PCR products using primers at the loci of *kae1-bud32* promoters (right). The schematic for the promoter replacement is shown on the left. 1, TsaKI; 2, TsaKI/P_{araS}*38::cgi121*. M, marker.

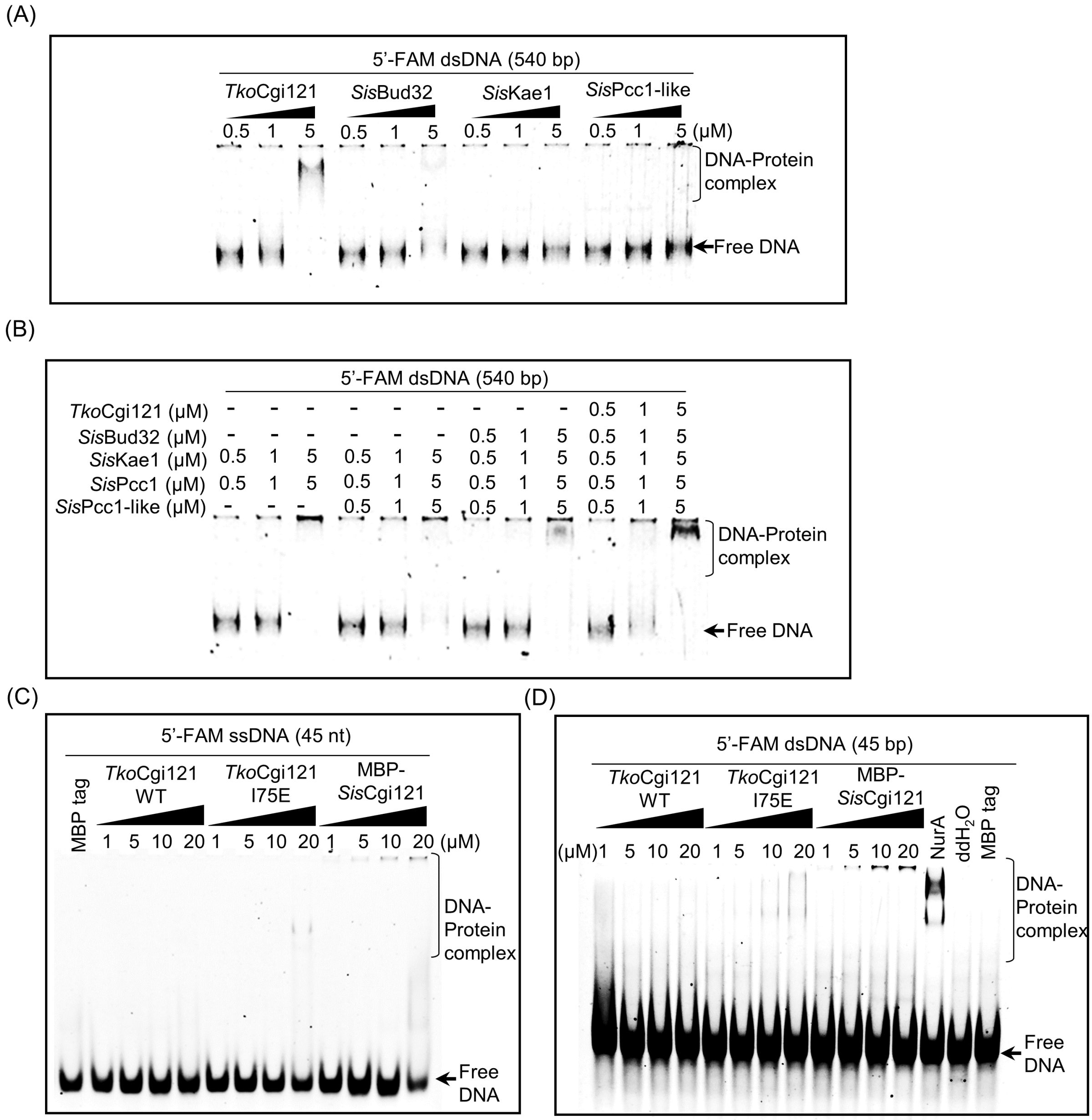

Figure S5. Analysis of the DNA binding ability of archaeal KEOPS. (**A**) Binding of individual subunit for dsDNA. (**B**) Binding of combinations of the subunits for dsDNA. FAM-labeled dsDNA (10 nM) was incubated with samples of the indicated subunits (0.5, 1, 5 μM) at 37C° for 30 min. (**C**) *Tko*Cgi121 (WT), the CCA-3' RNA binding site mutant *Tko*Cgi121 (I75E), and *Sis*Cgi121 for ssDNA binding. Purified Cgi121 (1, 5, 10, 20 μM) was incubated in a reaction mixture containing 10 nM of FAM-labeled ssDNA（45 nt, with two CCA motifs colored in red）at 37C° for 45 min. *Sis*SSB (5 μM) was used as a positive control and MBP-tagged *Sis*Pcc1 (20 μM) was used as negative control. (**D**) *Tko*Cgi121 (WT), *Tko*Cgi121 (I75E), and *Sis*Cgi121 for short dsDNA binding. Purified Cgi121 (1, 5, 10, 20 μM) was incubated in a reaction mixture containing 10 nM of FAM-labeled dsDNA (45 bp, with two CCA motifs colored in red) at 37C° for 45 min. *Sis*NurA (5 μM) was used as a positive control and MBP-tagged *Sis*Pcc1 (20 μM) was used as negative control. The samples were loaded in native PAGE gels. The gels were scanned using Amersham ImageQuant 800 after electrophoresis. The detailed reaction conditions are described in the Materials and Methods.

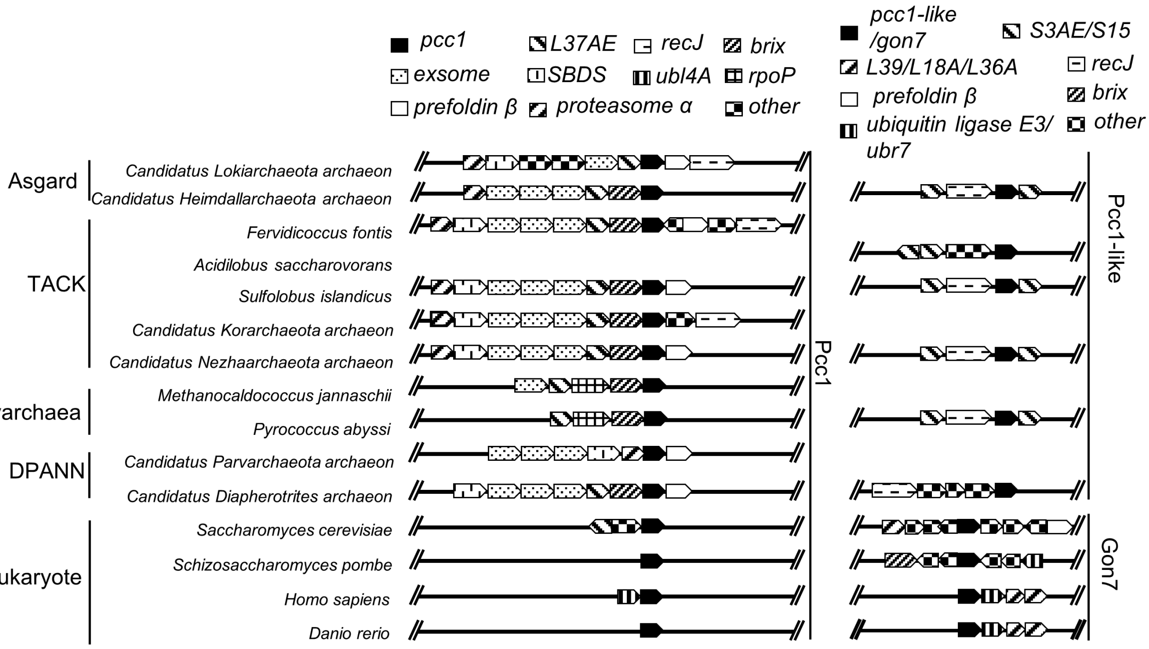

Figure S6. Genome context analysis of *pcc1*, *pcc1-like*, and *gon7* in archaea and eukaryotes. Representative species possessing one *pcc1* and two *pcc1* paralogs are shown for each superphylum of archaea together with several eukaryotic species. The *pcc1-like* occurred possibly via gene duplication of *pcc1* and was separated together with *recJ* during evolution.

Table S1. Strains used in this study

Strain	Properties	Source or reference
S. islandicus E233S	*S. islandicus* REY15A Δ*pyrEF* Δ*lacS*	Peng *et al.*, 2009, 2012
E233S/P_{araS}*24::kae1-bud32*	*kae1-bud32* promoter replaced with P_{araS}*24* in E233S	this study
E233S/P_{araS}*38::kae1-bud32*	*kae1-bud32* promoter replaced with P_{araS}*38* in E233S	this study
E233S/P_{araS}*24::cgi121*	*cgi121* promoter replaced with P_{araS}*24* in E233S	this study
E233S/*amyα::TmTsaB/TsaE/TsaC*	*TmTsaBEC* knocked in *amyα* (*sire_1098)* in E233S	this study
TsaKI	*TmTsaD* knocked in *mannanase* (*sire_2238*) in E233S/*amyα::TmTsaBEC*	this study
TsaKI/P_{araS}*38::kae1-bud32*	*kae1-bud32* promoter replaced with P_{araS}*38* in TsaKI	this study
E233S/P_{araS}*38::cgi121*	*cgi121* promoter replaced with P_{araS}*38* in TsaKI	this study

Table S2. Vectors used in this study.

Vectors	Properties or usages	Source or reference
pGE	*Sulfolobus-E. coli* shuttle vector containing mini-CRISPR and *pyrEF* for CRISPR-Cas based gene editing	Li *et al.*, 2016
pGE-*kae1*mg-KD	knock down of *kae1* with multi-gRNA	this study
pGE-*bud32*mg-KD	knock down of *bud32* with multi-gRNA	this study
pGE-*pcc1*sg-KD	knock down of *pcc1* with single-gRNA	this study
pGE-*pcc1-like*sg-KD	knock down of *pcc1-like* with single-gRNA	this study
pGE-*kae1*-KO	knock out of *kae1* with single-gRNA	this study
pGE-*bud32*-KO	knock out of *bud32* with single-gRNA	this study
pGE-*kae1-bud32*-KO	knock out of kae1-*bud32* with multi-gRNA	this study
pGE-*cgi121*-KO	knock out of *cgi121* with single-gRNA	this study
pGE-*pcc1*-KO	knock out of *pcc1* with single-gRNA	this study
pGE-*pcc1-like*-KO	knock out of *pcc1-like* with single-gRNA	this study
pGE-*cgi121*M52E	construction of *cgi121* mutant M52E with single-gRNA	this study
pGE-*cgi121*I64E	construction of mutant *cgi121* mutant I64E with single-gRNA	this study
pGE-*TmTsaBDEC*-KI	*tsaBDEC* knock in at *amyα* locus with single-gRNA	this study
pGE-*TmTsaD*-KI	*TmtsaD* knock in at *mannanase* locus with single-gRNA	this study
pSeSD	*Sulfolobus-E. coli* shuttle vector containing *araS*-SD promoter and MCS for proteins expression in E233S	Peng *et al.*, 2012
pSeSD-His-*Sis*Kae1	expression of C-terminal 6×His tagged *Sis*Kae1	this study
pSeSD-His-*Sis*Bud32	expression of C-terminal 6×His tagged *Sis*Bud32	this study
pSeSD-His-*Sis*Bud32D134A	expression of C-terminal 6×His tagged *Sis*Bud32D134A	this study
pSeSD-His-*Sis*Cgi121	expression of N-terminal 6×His tagged *Sis*Cgi121	this study
pSeSD-His-*Sis*Pcc1	expression of C-terminal 6×His tagged *Sis*Pcc1	this study
pSeSD-His-*Sis*Pcc1-like	expression of C-terminal 6×His tagged *Sis*Pcc1-like expression	this study
pSeSD-*Siskae1*-(His)*bud32*	expression of *Siskae1-bud32* operon with bud32 being tagged with C-terminal 6×His	this study
pSeSD-2ParaS	containing two *araS* promoter for protein expression	this study
pSeSD-2ParaS-*Sis*Kae1/His-*Sis*Bud32	Co-expression of *Sis*Kae1 and C-terminal 6×His tagged *Sis*Bud32	this study
pSeSD-2ParaS-His-*Sis*Kae1/ His-*Sis*Bud32	Co-expression of C-terminal 6×His tagged *Sis*Kae1 and C-terminal 6×His tagged *Sis*Bud32	this study
pSeSD-2ParaS-His-*Sis*Cgi121/His-*Sis*Bud32	N-terminal 6×His tagged *Sis*Cgi121 and C-terminal 6×His tagged *Sis*Bud32 coexpression vector	this study

pSeGP	*araS-SD* replaced with P*gdhA* in pSeSD	this study
pSeGP-His-*Tm*TsaB	constitutive expression of *Tm*TsaB	this study
pSeGP-His-*Tm*TsaC	constitutive expression of *Tm*TsaC	this study
pSeGP-His-*Tm*TsaD	constitutive expression of *Tm*TsaD	this study
pSeGP-His-*Tm*TsaE	constitutive expression of *Tm*TsaE	this study
pET22b-His-*Sis*Cgi121	expression of the N-terminal 6×His tagged *Sis*Cgi121	this study
pET22b-His-*Sis*Cgi121	expression of C-terminal 6×His tagged *Sis*Cgi121	this study
pET15b-*Sis*Kae1	expression of no tagged *Sis*Kae1	this study
pET15b-*Sis*Bud32	expression of no tagged *Sis*Bud32	this study
pET15b-His-*Sis*Bud32	expression of N-terminal 6×His tagged *Sis*Bud32	this study
pRSFDuet1-His-*Sis*Kae1	expression of N-terminal 6×His tagged *Sis*Kae1	this study
pRSFDuet1-Flag-*Sis*Bud32	N-terminal Flag tagged *Sis*Bud32 expression vector	this study
pRSFDuet1-His-*Sis*Pcc1	N-terminal 6×His tagged *Sis*Pcc1 expression vector	this study
pRSFDuet1-His-*Sis*Pcc1-like	N-terminal 6×His tagged *Sis*Pcc1-like expression vector	this study
pRSFDuet1-His-*Sso*Cgi121	expression of N-terminal 6×His tagged *S. solfataricus* Cgi121	this study
pRSFDuet1-His-*Tko*Cgi121	expression of N-terminal 6×His tagged *T. kodakarensis* Cgi121	this study
pRSFDuet1-His-*Tko*Cgi121I75E	expression of N-terminal 6×His tagged *Tko*Cgi121(I75E)	this study
pRSFDuet1-His-*Sis*Pcc1/*Sis*Kae1	co-expression of N-terminal 6×His tagged *Sis*Pcc1 and no tagged *Sis*Kae1	this study
pRS Duet1-His-*Sis*Pcc1-like/Flag-*Sis*Kae1	co-expression of the N-terminal 6×His tagged *Sis*Pcc1-like and N-terminal Flag tagged *Sis*Kae1	this study
pRSFDuet1-Flag-*Sis*Pcc1/His-*Sis*Pcc1-like	co-expression of the N-terminal Flag tagged *Sis*Pcc1 and N-terminal 6×His tagged *Sis*Pcc1-like	this study
pMALc2X-MBP-His-*Sis*Cgi121	co-expression of the N-terminal MBP and C-terminal 6×His tagged *Sis*Cgi121	this study
pMALc2X-MBP-His-*Sis*Pcc1	co-expression of N-terminal MBP and C-terminal 6×His tagged *Sis*Pcc1	this study

Table S3. Oligonucleotide and promoter DNA sequences used in this study.

Oligonucleotides	Sequences (5'to3')
kae1-KO-Spacer	GGCGGAAATACAATCATAACTACCTTCTATAAAGGGAGGT
bud32-KO-Spacer	GCGGTGATCTAACAACTAACAATCTCATCCTAAGTTCTATA
kae1-bud32-KO-Spacer1	ACTATGAAAGGACAATATTAGAGGCTAAGATAATTTATAC
kae1-bud32-KO-Spacer2	GGCGGAAATACAATCATAACTACCTTCTATAAAGGGAGGT
kae1-bud32-KO-Spacer3	AGGAATATCTACCTCGTCTACTCTCCATCTAGGTCTTAT
cgi121-KO-Spacer	ACTATAATACAACTAGAAATAAGATAAAGAGTTCAACTAT
pcc1-KO-Spacer	ATAAAAACGAATTACAGGATATAATATATGATTCGATAAT
pcc1-like-KO-Spacer	ATTCAAACCAAGGTTGAAGGCAAAGAGTTAGAGATTGTAA
kae1-KD-Spacer1	TCAGAATGTATACTTTCTAAAGATCTTAGAAATATATGAA
kae1-KD-Spacer2	ATATCGTTATTTATAGAACTTAGGATGAGATTGTTAGTTG
kae1-KD-Spacer3	CACTGCAGGTACATTCACATCGTTTTTAAGCGCAGTATAA
bud32-KD-Spacer1	GGTCTTTGGCCTCAGTTGTTAAATACCCTATTTCAATATG
bud32-KD-Spacer2	CTACTGCTATATAATTAATATCATTTATACTAATATTAGC
bud32-KD-Spacer3	TATCCCTTTCGTTTGCCAATATGTAGGGTGGTTGATCTTT
pcc1-KD-Spacer	GGGATTTCTTAATTTTTACATATTTAGTATCTATCTTTTC
pcc1-like-KD-Spacer	TCGTTTGGCTTTGTAATTCTCATCTTTATTACAATCTCTA
kae1-bud32-PE-Spacer	AAGAAAAGATAAAATATGTTAGTACTGGGTATCGAATCTA
cgi121-PE-Spacer	TATCTAACTTACTTACTTGAACCTTCGATATCTCTAACCA
*cgi121*M52E/I64E-Spacer	TACTTCTCCTATTACCATATGAACAAATAAAGGATGCATT
1098-KI-Spacer	TTATTCTCAAGCTCTATTGGTAGCCATTTGAGAAATTCTA
2238-KI-Spacer	ATGCACCATCAGCACCGTAAACTAACGCTCCCCTCAGTAT
P_{araS}*24*	ATGTTAAACAAGTTAGGTATACTATTTATACCATAGTTAGGTCATAAAAGTACCCGAGACC
P_{araS}*38*	ATGTTAAACAAGTTAGTGATACTATTTATAAAATAGTTAGGTCATAAAAGTACCCGAGACC
P_{gdhA}(with 30bp *gdhA* leader)	(-556)AACACTAATGAGAAAGT……CCGGGAAAACGAATTTATATTG**ATG**GAAGAAGTTCTTAGTTCGAGTCTGCAT (+30) *
5'-FAM labled ssDNA	CAGTATGCTGCGTGAGGAATGATCCCATGACGTAACCACAGTGCC
kae1-qPCR-F	CTCGGAGTAGGAATAGCAAAAGATCA
kae1-qPCR-R	CGTTTTAGTAGATCTCCAGGCTTCAT
bud32-qPCR-F	GAGGCTAAGATAATTTATACTGCGCTT
bud32-qPCR-R	CCTTAACTATCTCCCCTTCTATATATTC

cgi121-qPCR-F	GTCAAGTCTTACCTATCGTACCGTTT
cgi121-qPCR-R	CGGTAATAGGAGAAGTAAGAAAAACATAG
pcc1-qPCR-F	GTGAAGATAGAGATTTCAATTTATCCAGATAAT
pcc1-qPCR-R	CCTAGCTCTTGTAATTGATGGTGCA
pcc1-like-qPCR-F	TTACCAGAAGGAATGTCAATTCAAACCA
pcc1-like-qPCR-R	TTATAGCATCAAATGACGATTGAAGTGC
1715-qPCR-F	TAACGGTGTCAGAGATAACCTACAC
1715-qPCR-R	TGTTATGTCCTTTACTCTTATACCTACC
tbp-qPCR-F	CAACAGTTACGTTAGAGCAAAGTTTGG
tbp-qPCR-R	GTAACCTTGGGCTGTTCTAATCTGA

* The numbers in the brackets indicate the position of the last nucleotide at the upstream (-) or downstream (+) of the start codon (in red).

Reference

Peng N., Xia, Q., Chen, Z., Liang, Y.X., and She, Q. (2009). An upstream activation element exerting differential transcriptional activation on an archaeal promoter. Mol Microbiol 74, 928-939.

Peng, N., Deng, L., Mei, Y., Jiang, D., Hu, Y., Awayez, M., Liang, Y., She, Q. (2012). A synthetic arabinose-inducible promoter confers high levels of recombinant protein expression in hyperthermophilic archaeon Sulfolobus islandicus. Appl Environ Microbiol, 78(16), 5630-5637. doi:10.1128/AEM.00855-12

Li, Y., Pan, S., Zhang, Y., Ren, M., Feng, M., Peng, N., Chen, L., Liang, Y.X., and She, Q. (2016). Harnessing Type I and Type III CRISPR-Cas systems for genome editing. Nucleic Acids Res 44, e34.
